# Strain- and Dose-Dependent Reduction of *Toxoplasma gondii* Burden in Pigs Is Associated with Interferon-Gamma Production by CD8^+^ Lymphocytes in a Heterologous Challenge Model

**DOI:** 10.3389/fcimb.2017.00232

**Published:** 2017-06-08

**Authors:** Malgorzata Jennes, Stéphane De Craeye, Bert Devriendt, Katelijne Dierick, Pierre Dorny, Eric Cox

**Affiliations:** ^1^Laboratory for Immunology, Faculty of Veterinary Medicine, Ghent UniversityMerelbeke, Belgium; ^2^National Reference Laboratory for Toxoplasmosis, Operational Direction Communicable and Infectious Diseases, Scientific Institute of Public Health, Security of Food Chain and EnvironmentBrussels, Belgium; ^3^Department of Biomedical Sciences, Institute for Tropical MedicineAntwerp, Belgium; ^4^Laboratory for Parasitology, Faculty of Veterinary Medicine, Ghent UniversityMerelbeke, Belgium

**Keywords:** *Toxoplasma gondii*, infection, pigs, IFN-γ, T lymphocytes, immunity

## Abstract

*Toxoplasma gondii* is a worldwide prevalent parasite of humans and animals. The global infection burden exceeds yearly one million disability-adjusted life years (DALY's) in infected individuals. Therefore, effective preventive measures should be taken to decrease the risk of infection in humans. Although human toxoplasmosis is predominantly foodborne by ingestion of tissue cysts in meat from domestic animals such as pigs, the incidence risk is difficult to estimate due to the lack of screening of animals for infection and insights in location and persistence of the parasite in the tissues. Hence, experimental infections in pigs can provide more information on the risk for zoonosis based on the parasite burden in meat products intended for human consumption and on the immune responses induced by infection. In the present study, homo- and heterologous infection experiments with two distinct *T. gondii* strains (IPB-LR and IPB-Gangji) were performed. The humoral and cellular immune responses, the presence of viable parasites and the parasite load in edible meat samples were evaluated. In homologous infection experiments the parasite persistence was clearly strain-dependent and inversely correlated with the infection dose. The results strongly indicate a change in the amount of parasite DNA and viable cysts in porcine tissues over time. Heterologous challenge infections demonstrated that IPB-G strain could considerably reduce the parasite burden in the subsequent IPB-LR infection. A strong, however, not protective humoral response was observed against GRA7 and TLA antigens upon inoculation with both strains. The *in vitro* IFN-γ production by TLA-stimulated PBMCs was correlated with the infection dose and predominantly brought about by CD3^+^CD4^−^CD8α^bright^ T-lymphocytes. The described adaptive cellular and humoral immune responses in pigs are in line with the induced or natural infections in mice and humans. Previous studies underscored the heterogeneity of *T. gondii* strains and the corresponding virulence factors. These findings suggest the potential of the IPB-G strain to elicit a partially protective immune response and to reduce the parasite burden upon a challenge infection. The IPB-G strain could be used as a promising tool in limiting the number of viable parasites in edible tissues and, hence, in lowering the risk for human toxoplasmosis.

## Introduction

Toxoplasmosis is a parasitic infection caused by the intracellular protozoa *Toxoplasma gondii*. This parasite has a complex lifecycle and affects its definitive host as well as various intermediate hosts, among which domestic and wild animals and humans (Dubey, 2010). During its distinct developmental phases, the parasite manifests itself as a tachyzoite, a bradyzoite or a sporozoite in the oocyst. The sexual multiplication proceeds only within the definitive hosts (domestic or wild members of the family *Felidae*), wherein five morphologically different generations of the gamonts develop in the enterocytes, leading to formation of the gametocytes. Following the fertilization of the micro- and the macrogamete and the rupture of the infected cell, the unsporulated oocysts are discharged into the intestinal lumen. The final hosts are responsible for the extensive shedding of oocysts in the environment (Dubey, 1995; Afonso et al., 2008). The sporulated oocysts containing eight sporozoites show a high resistance to different environmental factors and under convenient circumstances may remain infectious for 1.5 years (Dubey, 2010). On average, the final host sheds at least one million oocysts in the acute phase of the infection, resulting in a massive contamination of the environment. This explains the persistence of the parasite in wild reservoir and livestock (Black and Boothroyd, 2000; Afonso et al., 2008; Innes, 2010; Opsteegh et al., 2016). The sporulated oocysts release the sporozoites upon ingestion by the intermediate host, followed by a differentiation to tachyzoites, several cell divisions in the enterocytes, and eventually dissemination to the peripheral tissues. There, the fast-multiplying tachyzoites convert into tissue cysts with the slowly dividing bradyzoites, and remain there as the dormant stage of the infection. The predation of the intermediate host or its tissues by the Felidae leads to the new sexual reproduction cycle, in which the bradyzoites transform back to tachyzoites and merozoites (Dubey, 1995; Afonso et al., 2008).

Several infection routes have been described for the different hosts of *T. gondii*; the majority of the herbivorous animals acquires the infection through ingestion of water or plants contaminated with oocysts. The predation of other mammals or birds or ingestion of the placenta and/or the aborted offspring of small ruminants facilitates the transmission of toxoplasmosis to carnivores and omnivores (Black and Boothroyd, 2000; Innes, 2010). In humans, foodborne toxoplasmosis mainly results from the consumption of raw or undercooked meat from infected animals, like domestic pigs. The global prevalence of this parasite includes one third of the human population and as such represents one of the most common parasitic zoonosis worldwide (Tenter et al., 2000; Ajzenberg et al., 2002; Aspinall et al., 2002; Bosch-Driessen et al., 2002; Kijlstra and Jongert, 2008; Innes, 2010; Robert-Gangneux and Dardé, 2012; Torgerson and Mastroiacovo, 2013). Consequently, infection in human has a severe short- and long-term impact, ranging from congenital or adult toxoplasmosis in healthy individuals to *T. gondii*-induced encephalitis in immune-compromised patients. In particular congenital toxoplasmosis in seronegative pregnant women has a very severe clinical relevance for the fetus, since the infection may result in abortion, intracranial calcifications, mental retardation or chorioretinitis in the newborn (Peyron et al., 2017). Finally, acquired toxoplasmosis has recently been associated with an increase in suicide rates or Parkinson's disease (Israelski and Remington, 1993; Holland, 2003; Lester, 2010; Miman et al., 2010; Wang et al., 2017). Therefore, numerous preventive measures are recommended in an attempt to decrease the global infection burden in the human population. The commonly applied precautions include hygienic processing of water and meat, such as boiling of surface water and avoiding the consumption of raw or undercooked meat, as only long term freezing at −12°C or baking above 67°C can effectively deactivate tissue cysts. Especially, pork is often consumed undercooked and is processed in many other meat products, reaching on average 300 consumers per pig (Fehlhaber et al., 2003; Belluco et al., 2016). Additionally, direct contact with contaminated soil, plants or cat feces should be avoided by wearing gloves when gardening or emptying the litter box, and by thoroughly washing fresh vegetables and fruits. Providing clear information on these preventive measures to seronegative pregnant women, in combination with a frequent serological screening to detect the acute infection during pregnancy, has proven to be successful in decreasing the infection rate (Breugelmans et al., 2004; Peyron et al., 2017). In livestock, the preventive measures are predominantly focused on strictly indoor housing, preventing access for cats, rodent control and the appropriate carcass disposal (Jones and Dubey, 2012; Robert-Gangneux and Dardé, 2012). Whereas environmental contamination as well as the prevalence of toxoplasmosis in sheep is overall high, the situation in regard to pigs may vary per country or the farm management. Consequently, the lack of uniform validation of the variety of serological assays, and the missing gaps in the correlation between the persistence of antibodies and parasite in pork are still to be improved. Nevertheless, the estimated average prevalence in the pig population seems to be very low in European countries (2.2%) and the USA (2.7%), presumably due to a shift from small and less strictly confined to large scale facilities, implementing all-in-all-out or farrow-to-finish models (Hill et al., 2008; EFSA, 2012; Guo et al., 2015). However, the recent rise of organic or free-range farming in order to improve animal welfare seems to contribute to an increase in infection rate in pig livestock and, as such, to the incidence of foodborne human toxoplasmosis (Kijlstra et al., 2004; Dubey et al., 2012; EFSA, 2012). The risk for humans to become infected by consumption of undercooked or raw pork is also not clear. The knowledge of the parasite persistence in edible tissues of naturally infected pigs is limited, as are the role of strain or dose in the parasite survival in the host. Such information might be of pivotal importance for vaccine development.

In the last decades numerous potential vaccine candidates have been experimentally tested mainly in mice and to a lesser extent in pigs. The formulations varied between a single recombinant parasitic protein or a combination of antigens, among which surface antigens (SAGs) or excretion/secretion proteins (GRAs, ROPs, MICs), but also DNA vaccines encoding B or T cell epitopes have been evaluated. However, the degrees of success were variable and did not led to a commercial vaccine (Vercammen et al., 2000; Letscher-Bru et al., 2003; Jongert et al., 2008; Li et al., 2011; Cao et al., 2015; Wagner et al., 2015). Vaccination did move forward by the use of attenuated viable strains, which resulted with a single commercially available vaccine for sheep, but their efficiency is tested under experimental circumstances and strictly species-dependent, and cannot yet be extrapolated to other livestock species or humans (Katzer et al., 2014; Burrells et al., 2015). Nevertheless, several experimental data in pigs reported reduction in parasite burden in infected and subsequently heterologous challenged pigs, in which the choice of the strain had an important effect on the viability of the parasite (Solano Aguilar et al., 2001; Dawson et al., 2004, 2005; Kringel et al., 2004; Garcia et al., 2005; Verhelst et al., 2011, 2015). In these studies, the involvement of the innate and acquired immune system was observed, dominated by antibodies production against the parasitic antigens, and by the Th1-type of the immune response. Depending on the experimental model, high levels of anti-GRA7 alone or anti-GRA1, -GRA7 and –TLA IgG's were detected upon an inoculation with IPB-G strain or a DNA GRA1-GRA7 cocktail vaccination, followed by the RH-strain or IPB-G challenge, respectively (Jongert et al., 2008; Verhelst et al., 2011), whereas a challenge with heterologous M4-strain oocysts after an experimental inoculation with viable S48-strain tachyzoites elicited a high TLA-specific IgG production (Burrells et al., 2015). In addition to the enhanced humoral immunity, a polarized Th1-immune response was observed after inoculation with a variety of the infectious *T. gondii* strains (Solano Aguilar et al., 2001; Dawson et al., 2004, 2005; Jongert et al., 2008; Verhelst et al., 2015). The significantly increased IFN-γ protein concentration in serum and the supernatant from the cultured PBMCs, and IFN-γ mRNA or DNA expression in PBMCs and intestinal lymphoid tissues, appeared positively correlated with the duration of the experiments (Solano Aguilar et al., 2001; Dawson et al., 2004, 2005; Jongert et al., 2008; Verhelst et al., 2015). In parallel with IFN-γ, also other cytokines were involved in the immune response against the parasite, as shown in the infection with the VEG-strain oocysts and the increased secretion of IL-15 and TNF-α (Dawson et al., 2005). Subsequently, a Th-2 response profile with predominantly IL-10 as anti-inflammatory cytokine was observed after the early phase of the infection, dominated by IFN-γ production, as mentioned earlier (Solano Aguilar et al., 2001; Aliberti, 2005). In contrast, IL-12 (IL-12p35 and IL-12p40) mRNA expression was not detected in PBMCs shortly after inoculation (7 and 14 dpi) in another study in pigs (Dawson et al., 2005). Nonetheless, even an excessive production of parasite-specific antibodies or Th-1/Th2-response cytokines did not provide a full protection during the acute phase of the infection, preventing from the cysts formation. Despite the active role the different components of the host's immune system play in the early stage of *T. gondii* infection, it remains a subject of discussion and ongoing research, whether the intermediate host can clear the tissues from the cysts on long term. It is noteworthy, however, that several studies in pigs notified reduced or undetectable counts of the parasite DNA in multiple porcine tissues, and a decline in viability of the cysts, as tested by bio-assay in mice (Jongert et al., 2008; Verhelst et al., 2011, 2015; Burrells et al., 2015). Taking into account the lack of an obligatory screening of pigs or pork meat to prevent transmission to humans, knowledge on the pig as an intermediate host for *T. gondii*, and in particular strategies to reduce the amount of viable parasites in tissues, may contribute to diminishing the risk of zoonosis by consumption of porcine meat (EFSA, 2007, 2012; Opsteegh et al., 2016). In light of these data, the aim of this study was to confirm differences between *T. gondii* strains in persistence of the parasite in tissues of experimentally infected pigs and to relate the dose and strain to the immune responses in the pigs upon a single infection or a heterologous challenge.

## Materials and methods

### *T. gondii* strains

Two *T. gondii* strains were used for the experimental infections: the IPB-Gangji (IPB-G) strain and the IPB-LR strain. The first one was isolated from the placenta of a patient with congenital toxoplasmosis and is highly virulent in mice. It produces a large number of tissue cysts and has an atypical mixed type I and type II genotype (Ajzenberg et al., 2002). The latter was isolated from pigs and belongs to genotype II, which is less pathogenic and commonly present in the European pig population (Dubey, 2009; Dubey et al., 2012). Both strains are maintained at the National Reference Laboratory for Toxoplasmosis (Scientific Institute for Public Health, Brussels, Belgium) by passage in Swiss female mice, since there is no alternative available to obtain a sufficient number of tissue cysts for the inoculation experiments than via bio-assay, as approved by the Ethical Committee (no. 20140704-01) and conform the European legislation (2010/63/EU). Tissue cysts from both strains were isolated from homogenized brain tissue, counted by phase-contrast microscopy and suspended in 10 ml of sterile phosphate buffered saline (PBS) solution at the desired concentration (700 cysts for the low dose and 6,000 for the high dose). The animals were inoculated within 8 h after cysts isolation. The inoculum for the negative control group was prepared identically from naive Swiss mice.

### Animals and experimental design

Two-week-old Belgian Landrace piglets were tested for the presence of anti-*T. gondii* serum antibodies (IgM and IgG) with an indirect immunofluorescence assay (IFA) as described previously (Verhelst et al., 2015). For the infection experiments, 3-week-old newly weaned, seronegative piglets were selected and randomly assigned to 10 groups of 3 animals (Table 1). These groups were housed in isolation units (Biosafety permit no, AMV/11062013/SBB219.2013/0145) at the Faculty of Veterinary Medicine, Ghent University, Belgium. All experiments were approved by the Ethical Committee of the faculties Veterinary Medicine and Bioscience Engineering at Ghent University (EC 2009/149).

**Table 1:** Experimental design: G, IPB-G strain; LR, IPB-LR strain.

**Study no**.	**Strain**	**Dose of tissue cysts**	**Group**	**Number of animals**	**Duration (dpi)**	**Heterologous challenge (y/n)**
1	G	700	G_low_	3	120	No
	G	6000	G_high_	3	120	No
	LR	700	LR_low_	3	120	No
	LR	6000	LR_high_	3	120	No
2	G	6000	G_high/_ LR_high_	3	120	Yes (at 60 dpi)
	G	6000	G_high1/2t_	3	60	No
	LR	6000	LR_high/_ G_high_	3	120	Yes (at 60 dpi)
3	G	6000	G_high_	3	98	No
	LR	6000	LR_high_	3	98	No
Control	/	0	G+LR_cltr_	7	120	No

*Groups, a single low or high dose of the IPB-G strain (G_low_ and G_high_); a single low or high dose of the IPB-LR strain (LR_low_ and LR_high_); a high dose of the IPB-G strain, followed 60 dpi by a high dose of the IPB-LR strain (G_high_/LR_high_); a high dose of the IPB-G strain 60 dpi (G_high1/2t_); a high dose of the IPB-LR strain, followed 60 dpi by a high dose of the IPB-G strain (LR_high_/G_high_)*.

**Figure 1:**
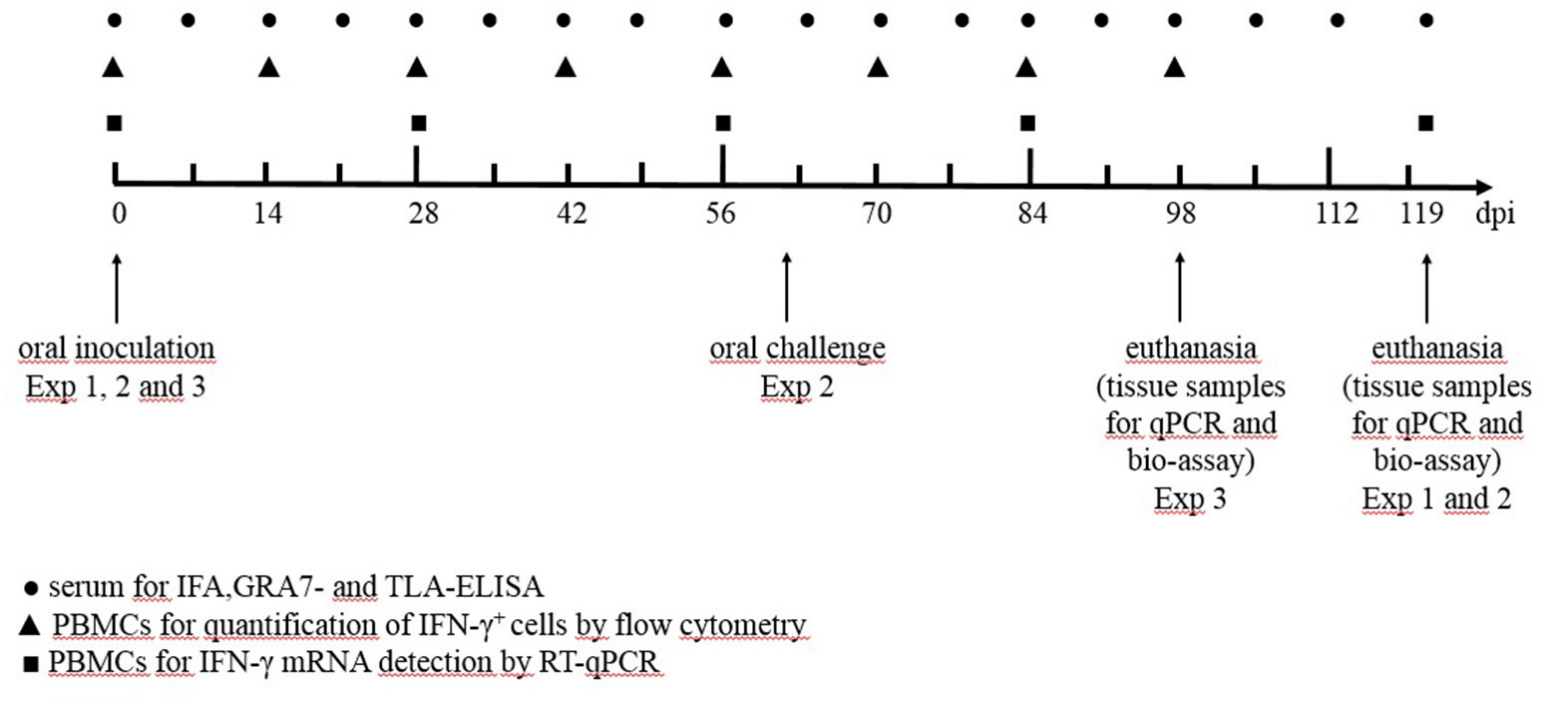
The timeline of the experiments from the inoculation (day 0 dpi) until euthanasia (day 120 dpi). Exp, experiment; ● serum for IFA, GRA- and TLA-ELISA; ▲ PBMCs for the quantification of IFN-γ^+^ T-lymphocytes by flow cytometry; ◼ PBMCs for IFN-γ mRNA detection by qPCR.

In a first experiment we aimed to study the effect of a low or high infection dose of two different *T. gondii* strains on the humoral and cellular immune responses and tissue cyst persistence until 120 days after inoculation (Table 1). In a second experiment we focused on the effect of a subsequent challenge with a heterologous strain at 60 dpi and the persistence of the parasite in the tissues at 120 dpi (Table 1). In study 3 we compared kinetics of the IFN-γ producing porcine T cell subsets following infection with high doses of the IPB-G or the IPB-LR strain until 98 dpi (Table 1). In each experiment the peripheral blood monomorphonuclear cells (PBMCs) were sampled at regular intervals for the detection of cytokine mRNA by RT-qPCR, and for the quantification of the IFN-γ producing T cell subsets, respectively. At euthanasia, PBMCs and lymphocytes from the peripheral lymph nodes and spleen were isolated for further *in vitro* assays, whereas heart, diaphragm, skeletal muscles and brain were collected to determine the parasite load as explained further. The experimental timeline presenting the collected samples and the sampling intervals is shown in Figure 1.

### Humoral immune response

For each experiment the seroconversion was monitored during the first 2 weeks after inoculation (wpi) by daily and subsequently weekly blood collection from the vena jugularis until 120 days post infection (dpi).

#### Antibody ELISA's with recombinant GRA7 and native TLA antigens

As dense granule protein 7 (GRA7) is considered as a marker of an active infection, being expressed by all *T. gondii* stages, recombinant GRA7 is frequently used to demonstrate the immune response during acute and chronic toxoplasmosis in humans and animals (Jacobs et al., 1999). GRA7 was prepared as previously described (Jongert et al., 2007). Briefly, GRA7 was produced as a His-tagged fusion protein by *Escherichia coli* (*E. coli*) TOP 10 cells (Life Technologies, Ghent, Belgium) and purified under denaturing conditions (8 M urea, 0.1% SDS) using nickel-nitrilotriacetic acid (Ni-NTA) chelate affinity column chromatography (Ni-NTA Superflow, Qiagen, Venlo, The Netherlands). GRA7 was then eluted from the Ni-NTA column using 250 mM imidazole and further purified by sequential dialysis steps reducing the urea and SDS concentration to 0.1 M and 0.01%, respectively.

*T. gondii* total lysate antigen (TLA) from tachyzoites of the RH-strain was prepared as previously described (Jongert et al., 2007) in the biosafety level 2 laboratory (Biosafety permit no, 415240), as approved by the Ethical Committee (no. 20140704-01) at the National Reference Laboratory for Toxoplasmosis (Scientific Institute for Public Health, Brussels, Belgium). TLA-based assays show a high reactivity due to a broad range of antigens in the lysate, however, differences in the production method can affect the composition of the lysate (Gamble et al., 2005; Ferra et al., 2015). Concisely, tachyzoites were diluted with PBS and then purified by differential centrifugation and filtration through a 5 μm syringe filter (MilleX®SV, Merck KGaA, Darmstadt, Germany). The tachyzoite suspension was then lysed by alternating sonication with cooling cycles using an Ultrasonic disintegrator (MSE, Leicester, United Kingdom). To evaluate the protein content of the lysate, the bicinchoninic acid (BCA) reaction (Thermo Scientific Pierce BCA protein Assay Kit, Erembodegem, Belgium) was used. Finally, the TLA was aliquoted and stored at −20°C until further use.

Both TLA and GRA7 were used in indirect Enzyme-Linked Immunosorbent Assays (ELISA's) at 10 μg/ml to detect *T. gondii*-specific IgM and IgG antibodies in serum samples diluted 1/50 with the goat anti-pig IgM- and IgG-Horse Radish Peroxidase (HRP) conjugate (Bethyl Laboratories Inc., Montgomery, Texas, USA), respectively, and 2,2′-azino-bis(3-ethylbenzothiazoline-6-sulphonic acid (ABTS) as substrate-chromogen solution (Verhelst et al., 2015). On each plate previously collected sera from one positive and three negative control animals as established by IgM and IgG immunofluorescence assay (IFA) were included and diluted 1/50 in dilution buffer (0.05% Tween-20 in PBS). The absorbance was measured at 405 nm (TECAN Spectra Fluor, Tecan Group Ltd., Männedorf, Switzerland) and the obtained data were analyzed in Microsoft Excel. Serum samples from infected animals were considered positive when exceeding the cut-off value calculated using the formula: mean OD_405_ negative controls + 3 x its standard deviation (SD).

#### Immunofluorescence assay

The presence of IgM and IgG antibodies against *T. gondii* was also evaluated by IFA using slides coated with formaline-fixed tachyzoites from the *T. gondii* RH-strain (Toxo-Spot IF, Biomérieux, Marcy-l'Etoile, France). Briefly, serum samples, diluted 1/50 in PBS, were applied to the slides for 30 min at 37°C, followed by washing with PBS. After drying, a second incubation with fluorescein isothiocyanate (FITC)-conjugated goat anti-swine IgM(μ) or IgG (H+L) (KPL, Maryland, USA) antibody (diluted 1/25 in PBS with Evans Blue as counter dye) was performed for 30 min at 37°C. After washing, drying and mounting with PBS-buffered glycerol, the slides were observed by fluorescence microscopy (Carl Zeiss, Germany). The cut-off read-out was established with positive and negative reference sera at a 1/50 dilution.

### Detection of the cellular immune response

PBMCs were isolated from 20 ml heparinized blood (LEO Pharma, Ballerup, Denmark) by density gradient centrifugation (800 × g at 18°C, 25 min) using Lymphoprep™ (Axis-Shield, Oslo, Norway) (Sonck et al., 2010). Subsequently, the cell pellets were resuspended in leukocyte medium [RPMI-1640 (GIBCO BRL, Life Technologies, Merelbeke, Belgium), supplemented with fetal calf serum (10%) (Greiner, Bio-One, Merelbeke Belgium), non-essential amino acids (100 mM) (Gibco), Na-pyruvate (100 μg/ml), l-glutamine (292 μg/ml) (Gibco), penicillin (100 IU/ml) (Gibco), streptomycin (100 μg/ml) (Gibco), and kanamycin (100 μg/ml) (Gibco)]. The cells (10^6^ cells/well) were cultured for 6 and 72 h upon stimulation with either TLA (10 μg/ml) as a heterologous challenge or the mitogen concanavalin A (ConA, Sigma-Aldrich, USA; 5 μg/ml) as a positive control.

#### Cytokine mRNA quantification by RT-qPCR

After 6 h of incubation with TLA, ConA or medium, the cells were lysed by adding 350 μl of RLT-buffer (Qiagen) supplemented with 1% β-mercaptoethanol (99%, Thermo Fisher Scientific, Aalst, Belgium) and stored at −80°C until RNA isolation. Total RNA extraction and conversion into cDNA was performed using the RNeasy kit (Qiagen) and the iScript kit (Biorad, Hercules, CA, USA), respectively. The purity of the RNA was assessed by an on-column DNase digestion step as recommended by the supplier. The amount of cytokine cDNA was then tested by quantitative polymerase chain reaction (qPCR). The qPCR reaction mix consisted of 12.5 μl iQ SYBR Green Supermix (Biorad), 0.5 μl of each primer set at a concentration of 20 μM, 1.5 μl PCR grade water and 10 μl of the 1/100 diluted cDNA. Interleukin (IL)-10, IL-12A, IL-17A, and interferon-gamma (IFN-γ) cDNA was amplified with the primer sets presented in Table 2. In order to normalize the cytokine expression, β-actin, glyceraldehyde phosphate dehydrogenase (GAPDH) and the ribosomal 18S gene were used as reference genes (Table 2). Special care was taken to choose a set of primers on different exons or spanning exon-exon junctions to exclude the amplification of genomic DNA. The qPCR amplification protocol consisted of an initial denaturation at 95°C for 3 min, followed by 45 cycles of 95°C for 15 s and 61°C for 20 s. After each run, a melt curve analysis was performed to confirm the presence of the correct amplicon and to exclude false positives due to the formation of primer dimers. The cDNA was tested in duplicate for each cytokine and the three reference genes (GADPH, β-actin, r18S), showing a stable expression. The mRNA expression in PBMCs was calculated with the CFX96 Manager™ Software v3.1 (Biorad), using a mathematical model (delta-delta Ct method). The mean value was determined for the target cytokines and normalized relative to the geometric mean of the reference genes (Verhelst et al., 2015).

**Table 2:** List of primers for qPCR.

**Target**	**Sequence**	**Length amplicon (bp)**
IL-10	F: CCTGGGTTGCCAAGCCTT	240
	R: GCTTTGTAGACACCCCTCTCTT	
IL-12A	F: ACCAGCACAGTGGAGGC	95
	R: CGAATGAGAGTTGCCTGGCT	
IL-17A	F: GGACAAGAACTTCCCTCAGCA	124
	R: CTCGTTGCGTTGGAGAGTC	
IFN-γ	F: GAGCCAAATTGTCTCCTTCTACTT	262
	R: CTGACTTCTCTTCCGCTTTCT	
GAPDH	F: CCATCACTGCCACCCAGAA	130
	R: CAGGGATGACCTTGCCCA	
B-actin	F: GGCATCCTGACCCTCAAGTA	137
	R: GCCTCGGTCAGCAGCA	
r18S	F: GTTGATTAAGTCCCTGCCCTTT	141
	R: GATAGTCAAGTTCGACCGTCTT	

#### Flow cytometric detection of IFN-γ production

The flow cytometric detection of IFN-γ-producing proliferating lymphocytes was performed on cultured PBMCs 72 h after heterologous stimulation with TLA (10 μg/ml). First, the cell division marker Violet Proliferation Dye 450 (VPD450, BD Biosciences, Erembodegem, Belgium) was added to the isolated mononuclear cells, showing a diminishing fluorescence after each cell division. At the end of the incubation period, a protein transport inhibitor, Golgi Plug™, was added and the cells were fixed and permeabilized using the Cytofix/Cytoperm™ kit (both from BD Biosciences). Subsequently, cells were stained using murine monoclonal antibodies (Mab) against CD3 (IgG1, clone PPT3), CD4 (IgG2b, clone 72–14-4), and CD8 (IgG2a, clone 11/295/33) and anti-isotype-specific conjugates (goat anti-mouse IgG1-PerCP-Cy5.5; Santa Cruz Biotechnology, Dallas, Texas, USA), goat anti-mouse IgG2b-FITC (Southernbiotech, Birmingham, Alabama, USA) and goat anti-mouse IgG2a-Alexa Fluor®647 (Invitrogen™, Merelbeke, Belgium). Finally, phycoeryrthrin (PE)-conjugated mAb against porcine IFN-γ (Mouse IgG1, BD Biosciences) was added to identify the lymphocyte subsets producing IFN-γ. A minimum of 10,000 events was recorded within the proliferating cell gate (Appendix 1). The IFN-γ secretion in the different lymphocyte subsets was determined and compared with the results of the isotype-matched control (Mouse IgG1-PE, Abcam, Cambridge, UK) using a FACSAria III and FACSDIVA™ software (both from BD). The gating strategy is included in the supplementary data (Appendix 1).

Animals were euthanized at 98 dpi and the splenocytes and lymphocytes from the peripheral lymph nodes (mediastinal, mesenteric and popliteal) were isolated as previously described (Verhelst et al., 2011). Subsequently, the cells were stimulated with the same antigens as the PBMCs for 6 h and 72 h, whereafter the same staining occurred for flow cytometric analysis as for the PBMCs.

### Detection of the parasite: bio-assay and qPCR

In experiments 1 and 2, all animals were euthanized at 120 dpi and the parasite load was determined in brain (Br), heart (He), spleen, diaphragm (Di) and skeletal muscles (m. gastrocnemius (Mg), mm. intercostales (Ic), m. longissimus dorsi (Ld), and m. psoas major (Mp) by qPCR and a bio-assay. For this, 100 g of each tissue was homogenized in 10 ml 0.85% sodium chloride (NaCl) and digested with pepsin [0.8 g/l pepsin in 7 ml/l hydrogen chloride (HCl)] for 1 h for brain and 2 h for the other tissues, while stirring in a water bath at 37°C. The obtained suspension was filtered and centrifuged for 15 min at 1,180 × g, the supernatant removed and the pellet resuspended in 10 ml PBS supplemented with 40 IU/ml gentamicin. For the bio-assay, 1 ml of the tissue suspension was inoculated intraperitoneally into 5 naive Swiss female mice. The mice were observed frequently on a daily base for the next 5 weeks and euthanized in respect to the human end points in case of acute toxoplasmosis associated with suffering or reduced welfare. The surviving mice were euthanized and tested serologically by immunofluorescence for the presence of *T. gondii* IgG antibodies or by qPCR for lungs and ascites when pre terminated due to the ethical aspects in case of acute toxoplasmosis. To determine the parasite load by qPCR DNA was extracted from the tissue suspensions with the QIAamp DNA Mini kit (Qiagen). A 10-fold serial dilution of *T. gondii* DNA prepared from RH-strain tachyzoite suspension containing 10^6^ parasites per ml was used as a standard, with a detection limit of 2–4 tissue cysts per 100 g of tissue. Real-Time PCR (RT-PCR) amplifying both the *T. gondii* repeat element (AF146527) and the ribosomal 18S rDNA of the host cells was performed as previously described (Rosenberg et al., 2009).

### Statistics

The parasite-specific antibody and IFN-γ responses in different groups at different time points are presented as means ± SD. A one-way Analysis of Variance (ANOVA) was performed, followed by post hoc Bonferroni's and Dunnett's Multiple Comparison Tests for antibody production and cytokine response, respectively, to discriminate between infected and control groups (GraphPad Prism 5). A *p* < 0.05 was considered statistically significant.

## Results

### Parasite burden and immune response after single inoculation with a low or a high infection dose of the IPB-G or IPB-LR strain

#### GRA-7 and TLA-specific antibody response

**Figure 2:**
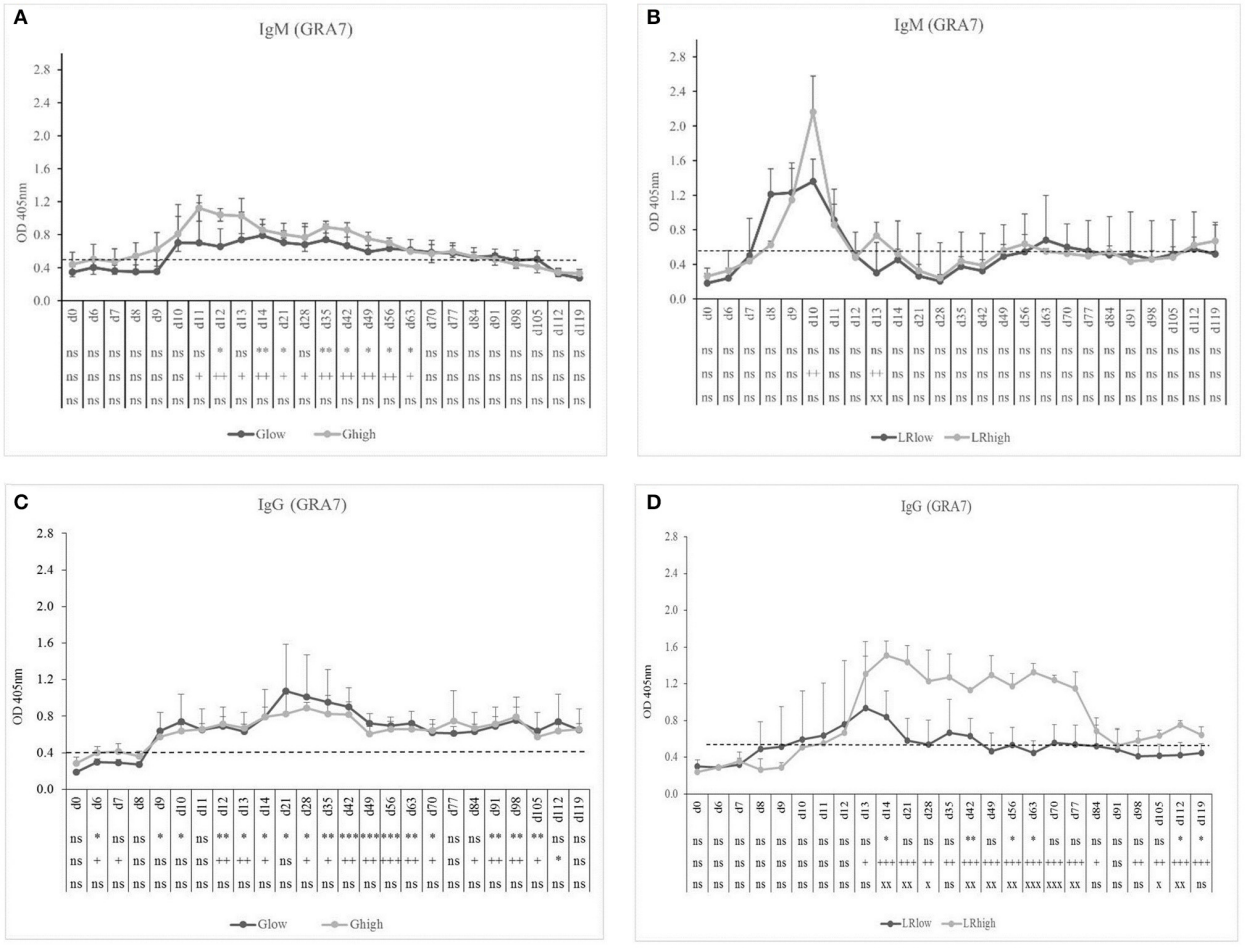
GRA7-specific IgM and IgG responses after inoculation with IPB-G or IPB-LR *T. gondii* strain. IgM **(A,B)** and IgG **(C,D)** responses in animals inoculated with a low or a high dose of the IPB-G **(A,C)** or the IPB-LR **(B,D)** strain. Groups: G_low_, G_high_, LR_low_, LR_high_. The horizontal dashed line indicates the cut-off value based on the mean of the negative animals. The results represent a mean of the infected group ± SD; ^*^ (low dose vs. controls) or + (high dose vs. controls) or x (low dose vs. high dose): *P* < 0.05, ^**^ or ++ or xx: *P* < 0.01; ^***^ or + + + or xxx: *P* < 0.001; ns, not significant.

The GRA7-specific IgM antibodies appeared approximately at the same time in the low and the high dose group (10 and 9 dpi, respectively) upon inoculation with the IPB-G strain and declined gradually from 14 and 11 dpi, respectively, until 91 dpi (Figure 2A). In contrast, in the IPB-LR infected group a pronounced IgM production was detected 8 dpi in the low dose group and even a stronger response at 10 dpi in the high dose group (Figure 2B), but both declined to control levels around 12 dpi. GRA7-specific IgG antibodies were detected shortly after IgM, irrespective of the inoculation strain, and remained detectable until the end of the experiment (Figures 2C,D). Nevertheless, the high dose of the IPB-LR strain induced the highest levels of GRA7-specific IgG.

**Figure 3:**
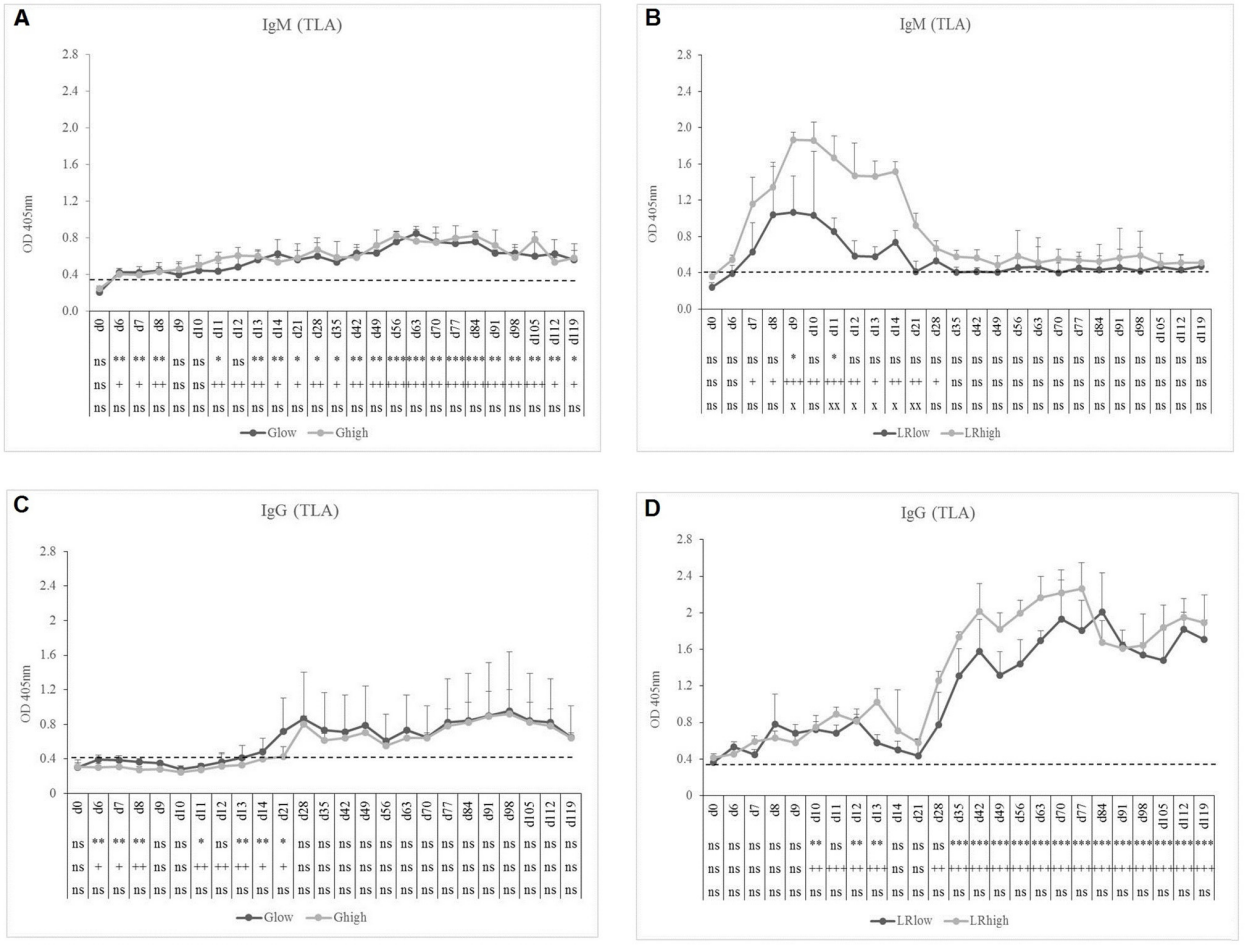
TLA-specific IgM and IgG responses after inoculation with IPB-G or IPB-LR *T. gondii* strain. IgM **(A,B)** and IgG **(C,D)** responses in animals inoculated with a low or a high dose of the IPB-G **(A,C)** or the IPB-LR **(B,D)** strain. Groups: G_low_, G_high_, LR_low_, LR_high_. The horizontal dashed line indicates the cut-off value based on the mean of the negative animals. The results represent a mean of the infected group ± SD; ^*^ (low dose vs. controls) or + (high dose vs. controls) or x (low dose vs. high dose): *P* < 0.05, ^**^ or ++ or xx: *P* < 0.01; ^***^ or +++: *P* < 0.001, ns, not significant.

TLA-specific IgM occurred earlier than GRA7-specific IgM, namely 7 to 8 dpi in the G_low_ and G_high_ groups. In the latter, the IgM response remained present until the end of the experiment, slightly increasing in time, irrespective from the infection dose (Figure 3A). On the contrary, for the LR_low_ and LR_high_ groups the seroconversion to TLA-specific IgM was prominently present until 21 to 28 dpi, showing again the highest concentration in the high dose group (Figure 3B). The TLA-specific IgG antibodies appeared approximately 14 dpi in both dose groups inoculated with the IPB-G strain (Figure 3C), but already at 8 dpi in animals infected with the IPB-LR strain. There, the antibodies increased significantly starting from 28 dpi and remained elevated until 120 dpi (Figure 3D). In animals inoculated with the IPB-G strain no dose effect was neither seen for TLA-specific IgM nor for IgG production (Figures 3A,C), whereas the high dose induced a higher response for both IgM and IgG upon inoculation with the IPB-LR strain (Figures 3B,D). The IFA results confirmed the seroconversion from *T. gondii*- negative toward IgM positive animals and the persistence of the IgG antibodies in each infection experiment (data not shown).

#### TLA-specific IFN-γ mRNA responses in PMBCs

**Figure 4:**
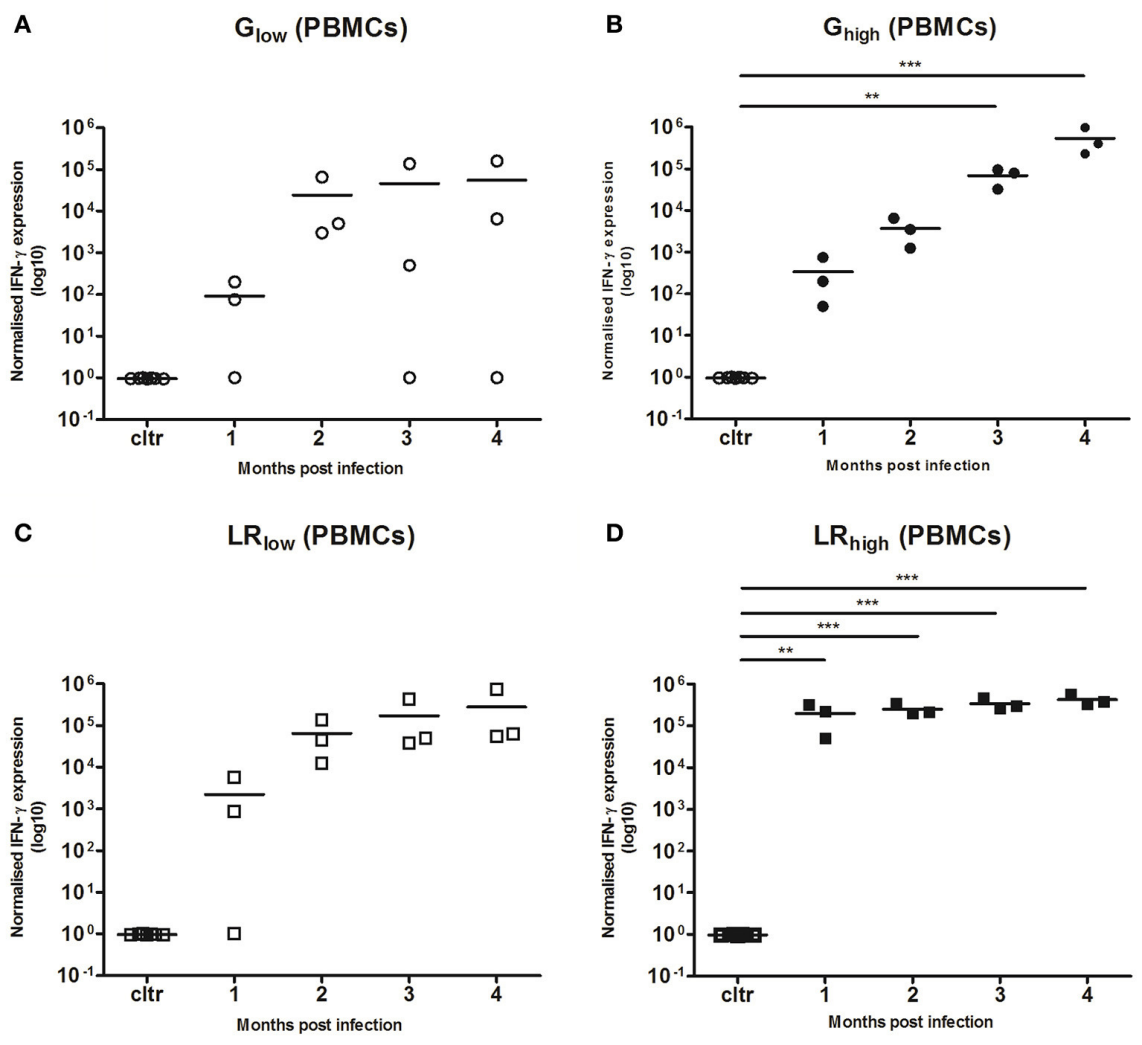
Relative normalized IFN-γ expression in PBMCs after a single IPB-G or IPB-LR inoculation. PBMCs were isolated monthly from pigs orally infected with **(A)** a low or **(B)** a high dose of the IPB-G strain (G_low_ and G_high_) or **(C)** a low or **(D)** a high dose of the IPB-LR strain (LR_low_ and LR_high_). Cells were restimulated *in vitro* with TLA and IFN-γ mRNA was quantified with RT-PCR. The thick lines indicate the group mean. The significance level: ^**^P < 0.01; ^***^P < 0.001.

PBMCs were restimulated *in vitro* with TLA for 6 h, where after IL-10, IL-12A, IL-17A, and IFN-γ mRNA responses were determined. No detectable IL-10, IL-12, and IL-17A mRNA production was observed (data not shown) in any infected group, irrespective of the strain or infection dose. However, a substantial increase in IFN-γ mRNA production was observed from 1 month post infection (mpi) onwards in the majority of the inoculated animals as compared to the control animals. This response was least pronounced in the animals infected with the low dose of the IPB-G (not significant, *p* = 0.39) (Figure 4A), followed by a significant (*p* < 0.01) and highly significant (*p* < 0.001) increase in the high dose of the IPB-G strain group (Figure 4B). In the low dose of the IPB-LR group we noticed a steady though not significant (*p* = 0.18) increase (Figure 4C). The highest IFN-γ production was observed in the animals infected with the high dose of the IPB-LR strain starting from 1 mpi (*p* < 0.01), which stayed high throughout the experiment, becoming highly significant (*p* < 0.001) at 2, 3, and 4 mpi (Figure 4D). No detectable IFN-γ level was detected in splenocytes from IPB-G or IPB-LR infected animals (data not shown).

#### Parasite load in tissues

At 120 dpi, the parasite load was determined in heart and striated skeletal muscles by qPCR and a bioassay, while in brain by qPCR only. In animals infected with the IPB-LR strain, the highest parasite load was found in brain followed by heart (Table 3). Interestingly, the inoculation dose did not affect the distribution or load in the tissues. Besides brain and heart, also intercostal muscles and the longissimus dorsi were consistently positive in qPCR, whereas heart of all 6 animals was also positive in the bioassay.

**Table 3:** Parasite load (number of bradyzoites per 1E+08 cells) by qRT-PCR after inoculation with two *T. gondii* strains, in comparison with bio-assay (number of positive/total tested).

	**qPCR**							**Bio-assay**						
	**Br**	**Ha**	**Di**	**Ic**	**Mg**	**Ld**	**Mp**	**Br**	**Ha**	**Di**	**Ic**	**Mg**	**Ld**	**Mp**
G_low_	2/3	2/3	2/3	3/3	2/3	0/3	0/3	nt	2/3	1/3	1/3	0/3	0/3	0/3
Average	1.1E+05	8.5	20.6	44.6	3.0	0.0	0.0							
SD	1.1E+05	8.4	29.9	13.4	2.7	0.0	0.0							
G_high_	0/3	1/3	0/3	2/3	0/3	0/3	1/3	nt	2/3	1/3	0/3	0/3	0/3	0/3
Average	0.0	1.0	0.0	21.2	0.0	0.0	2.3							
SD	0.0	1.0	0.0	19.1	0.0	0.0	4.0							
LR_low_	3/3	3/3	3/3	3/3	2/3	3/3	3/3	nt	3/3	3/3	1/3	0/3	0/3	0/3
Average	378.78	169.63	8.46	23.45	52.40	28.67	189.07							
SD	94.86	228.70	12.25	17.52	45.38	29.94	244.88							
LR_high_	3/3	3/3	2/3	3/3	3/3	3/3	2/3	nt	3/3	1/3	1/3	0/3	1/3	1/3
Average	1339.97	592.25	7.82	29.4	35.0	72.6	67.8							
SD	322.83	281.15	11.61	30.5	8.6	28.5	68.0							
G_high_/LR_high_	3/3	3/3	3/3	2/3	1/3	0/3	2/3	nt	3/3	2/3	1/3	0/3	0/3	1/3
Average	489.07	70.66	19.22	16.7	8.1	0.0	66.3							
SD	614.04	51.02	17.93	22.3	14.1	0.0	102.2							
G_high1/2t_	2/3	3/3	1/3	0/3	1/3	1/3	0/3	nt	2/3	1/3	0/3	0/3	0/3	0/3
Average	1742.74	16.18	5.82	0.0	2.2	6.7	0.0							
SD	1570.94	26.09	10.07	0.0	3.9	11.6	0.0							
LR_high_/G_high_	3/3	3/3	1/3	0/3	1/3	0/3	1/3	nt	2/3	1/3	0/3	1/3	0/3	1/3
Average	5950.50	105.32	1.67	0.0	0.8	0.0	1.6							
SD	8708.45	128.54	2.90	0.0	1.5	0.0	2.7							

*Groups, a single low or high dose of the IPB-G strain (G_low_ and G_high_) 120 dpi; a single low or high dose of the IPB-LR strain (LR_low_ and LR_high_); a high dose of the IPB-G strain, followed 60 dpi by a high dose of the IPB-LR strain (G_high_/LR_high_); a high dose of the IPB-G strain 60 dpi (G_high1/2t_); a high dose of the LR strain, followed 60 dpi by a high dose of the IPB-G strain (LR_high_/G_high_). Tissues: Br, brain; Ha, heart; Di, diaphragm; Ic, intercostal m.; Mg, gastrocnemius m.; Ld, long. dorsi m.; Pm, poas major m.; nt, not tested*.

A different pattern was seen in pigs inoculated with the IPB-G strain (Table 3), where a clear effect of the inoculation dose on the parasite distribution and load in the tissues was found. When inoculated with the low dose, the parasite was present in more tissues and in higher amounts than when inoculated with the high dose. However, even when inoculated with the low dose, the longissimus dorsi and psoas major remained negative in all three animals in this group. Summarizing, animals inoculated with the high dose of IPB-G showed the lowest amount of *T. gondii* DNA in their tissues. Brain, gastrocnemius and the longissimus dorsi were negative, whereas for diaphragm and psoas major only one sample was positive in the bioassay and qPCR, respectively. These results strongly suggest a dose-dependent decreased burden of the IPB-G strain in the examined tissues following inoculation, pointing toward an immune-mediated reduction of the parasite load.

### Parasite tissue load and immune response in a subsequent infection model with two *T. gondii* strains

In order to assess if the low parasite load observed in some tissues after infection with the IPB-G strain was related to an immune response, in a second experiment animals were first infected with the high dose of one strain, followed 60 days later with the high dose of the other strain (Table 1). Since we hypothesized an effect of the inoculation with the IPB-G strain, an additional control group was included in the study inoculated with the high dose at 60 dpi and 60 days before euthanasia.

#### GRA-7 and TLA-specific antibody response

**Figure 5:**
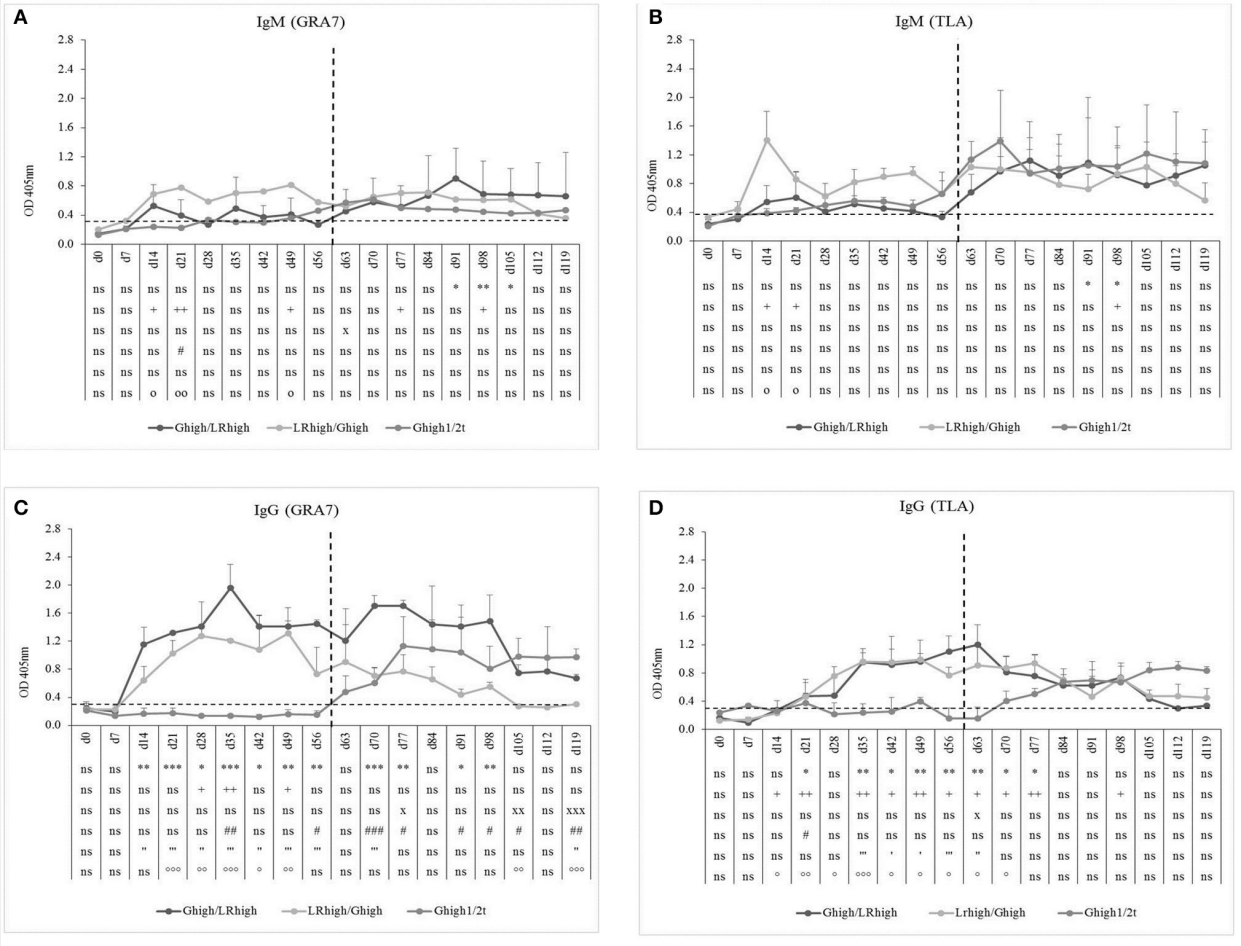
GRA7- and TLA-specific IgM and IgG responses after inoculation with IPB-G and IPB-LR *T. gondii* strains. IgM **(A,B)** and IgG **(C,D)** antibody response toward *T. gondii* GRA7 **(A,C)** and TLA **(B,D)** in animals after a consecutive infection with a high dose of IPB-G and IPB-LR strains. Groups: G_high_/LR_high_, LR_high_/G_high_. Piglets infected after 60 days with a high dose IPB-G served as a control (G_high1/2t_). The horizonthal dashed line indicates the cut-off value based on the mean of the negative animals, and the vertical one the timepoint of reinfection. The results represent a mean of the infected group ± SD; ^*^ G_high_/LR_high_ vs. controls, + LR_high_/G_high_ vs. controls, x G_high1/2t_ vs. controls, # G_high_/LR_high_ vs. LR_high_/G_high_, ' G_high_/LR_high_ vs. G_high1/2t_, ° LR_high_/G_high_ vs. G_high1/2t_: *P* < 0.05, ^**^ or ++ or xx or ## or “ or °°: *P* < 0.01; ^***^ or xxx or ### or ”' or °°°; *P* < 0.001; ns, not significant.

As in the first experiment, inoculation with the IPB-LR strain induced higher GRA7- and TLA-specific IgM production than with the IPB-G strain, independently from the order of inoculation (Figures 5A,B). This was most pronounced for the TLA-specific IgM response (Figure 5B). The presence of a clear TLA-specific IgM response after the second inoculation with the IPB-LR strain was remarkable and suggests differences in antigen expression between both strains (higher immunogenicity, different antigens or other reasons), leading to the induction of a primary immune response against various TLA antigens. Interestingly, the increase in TLA-specific IgM levels in the IPB-G infected animals upon initial inoculation at day 60 (G_high1/2t_) was higher in comparison with G_high_ or G_low_ groups (Figure 3A), suggesting maturation of the immune system. Similar to our findings of the first experiment (Figures 2C,D, 3C,D), the GRA7- and TLA-specific IgG antibodies appeared within 2 weeks following the primary inoculation with the IPB-G or IPB-LR strain (Figures 5C,D). A pronounced booster response against GRA7 occurred upon the heterologous challenge at 60 dpi in both re-infected groups, as evidenced by a much faster increase in IgG levels in contrast to animals from the G_high1/2t_ group (Figure 5C). The TLA IgG was not boosted following the heterologous infections in both challenged groups (Figure 5D). However, these distinct IgG responses were more pronounced by the challenge with the IPB-LR strain than with the IPB-G strain.

#### TLA-specific IFN-γ mRNA responses in PMBCs and spleen

**Figure 6:**
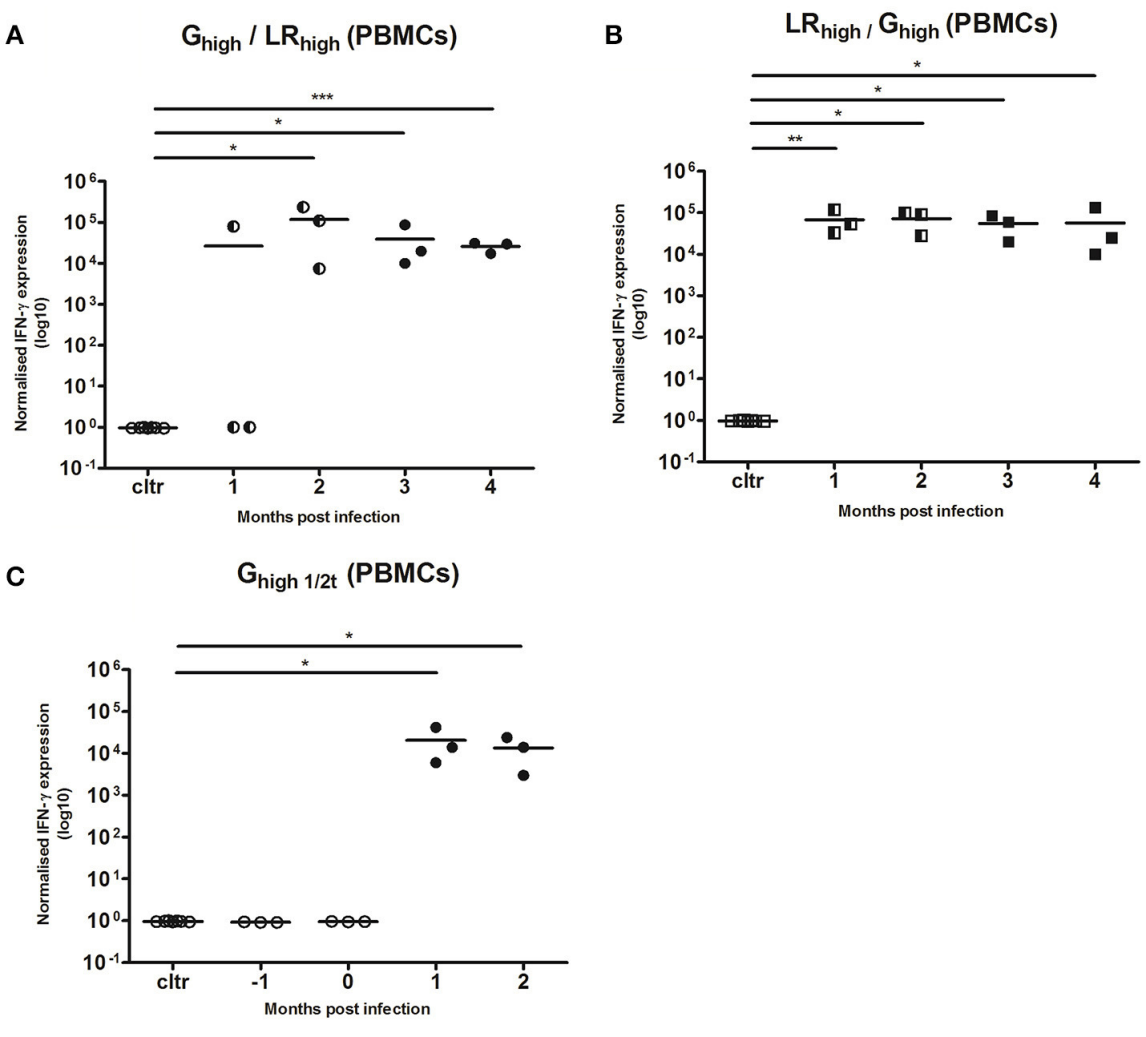
Relative normalized IFN-γ expression in PBMCs after a consecutive IPB-G and IPB-LR inoculation. PBMCs were isolated monthly from pigs orally infected with **(A)** a high dose of the IPB-G strain followed 60 days later by a high dose of the IPB-LR strain (G_high_/LR_high_) or **(B)** reversed infection model (LR_high_/G_high_). Piglets infected with a high dose of the IPB-G strain at day 60 served as a control **(C)** (G_high1/2t_). Cells were restimulated *in vitro* with TLA and IFN-γ mRNA was quantified with RT-PCR. The thick lines indicate the group mean. The significance level: ^*^P < 0.05, ^**^P < 0.01; ^***^P < 0.001.

IFN-γ responses after the initial inoculation with both *T. gondii* strains (Figure 6) were comparable with those in the first experiment (Figure 4). In two of the three animals receiving the IPB-G strain as a first inoculation (G_high_/LR_high_), IFN-γ mRNA expression could not be detected 1 mpi in the PBMCs recall assay with TLA. However, from 2 mpi all three animals showed a significantly (*p* < 0.05) to highly significant (*p* < 0.001) increased IFN-γ mRNA level (Figure 6A), similarly to the expression seen in animals receiving the IPB-LR strain as a first inoculum (LR_high_/G_high_) (Figure 6B), even though the latter showed a more homogenous response from 1 mpi onwards (*p* < 0.01). In both groups the IFN-γ mRNA expressions remained significantly elevated (*p* < 0.05 to *p* < 0.001) during the experiment and no additional increase was seen after inoculation with the heterologous strains. Slightly lower yet significant (*p* < 0.05) IFN-γ production was observed in group G_high1/2t_, inoculated for the first time at 60 dpi (Figure 6C).

**Figure 7:**
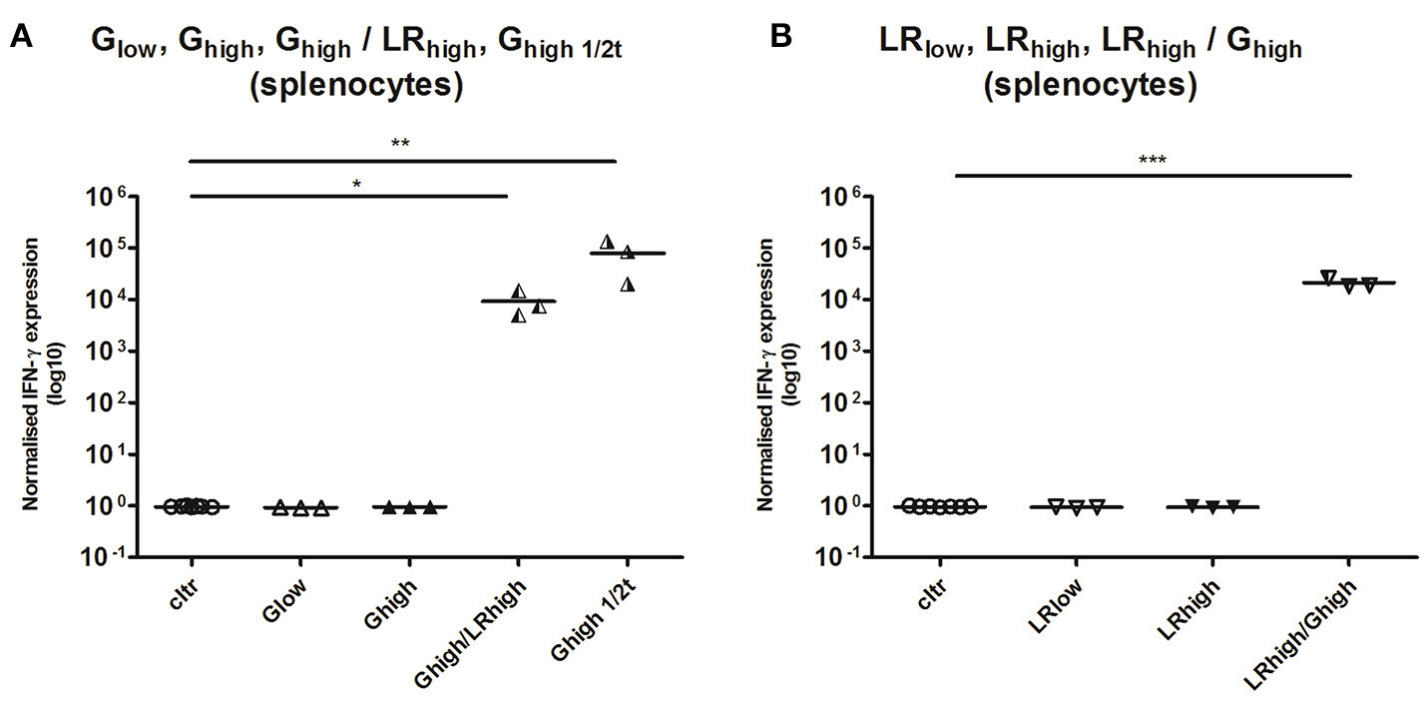
Relative normalized IFN-γ expression in splenocytes after a single or consecutive IPB-G and IPB-LR inoculation. Splenocytes were isolated from pigs orally infected with **(A)** a low dose of the IPB-G strain (G_low_), a high dose of the IPB-G strain (G_high_), a high dose of the IPB-G strain followed 60 days later by a high dose of the IPB-LR strain (G_high_/LR_high_), a high dose of IPB-G at day 60 (G_high1/2t_), or **(B)** a low dose of the IPB-LR strain (LR_low_), a high dose of the IPB-LR strain (LR_high_) or a high dose of the IPB-LR strain followed 60 days later by a high dose of the IPB-G strain reversed infection model (LR_high_/G_high_). Cells were isolated at 2 mpi (group: G_high1/2t_) or 4 mpi (groups: G_low_, LR_low_, G_high_, LR_high_, G_high_/LR_high_, LR_high_/G_high_) and restimulated *in vitro* with TLA. IFN-γ mRNA was quantified with RT-PCR. The thick lines indicate the group mean. The significance level: ^*^P < 0.05, ^**^P < 0.01; ^***^P < 0.001.

While no detectable cytokine production was found at 120 dpi in the spleen of animals from both infection groups in the first experiment (data not shown), in the heterologous infection model significant IFN-γ transcript levels were detected 60 days after the second infection for the G_high_/LR_high_ (*p* < 0.05), G_high1/2t_ (*p* < 0.01), and LR_high_/G_high_ (*p* < 0.001) (Figures 7A,B).

#### Parasite load in tissues

To assess the parasite load in tissues animals were euthanized 60 days after a first (G_high1/2t_ group) or second infection (G_high_/LR_high_ and LR_high_/G_high_ groups). Interestingly, animals receiving only the high dose of the IPB-G strain (G_high1/2t_) showed a parasite distribution and load (13 tissue samples negative in a bioassay) in between those observed at 120 dpi with the IPB-G low dose (10 tissue samples negative in a bioassay) and the high dose (17 samples negative) in the first experiment (Table 3). This could suggest that the reduction in parasite load induced by the IPB-G strain was already appearing at 60 dpi upon inoculation with the high dose. The tissue distribution at 120 dpi in animals, which first received the IPB-LR high dose and 60 days later the IPB-G high dose, could also be explained by this phenomenon. At 120 dpi the animals showed a different parasite load and tissue distribution (12 samples negative) than in the first experiment (1 and 2 samples negative after infection with the low and the high dose, respectively). Animals receiving first the IPB-G high dose and 60 days later the IPB-LR high dose, showed a wider parasite tissue distribution and a higher parasite tissue load (7 samples negative), more comparable to animals receiving only the IPB-LR strain.

### The involvement of CD4^+^ and CD8^+^ T cells in the strain-dependent IFN-γ production

**Figure 8:**
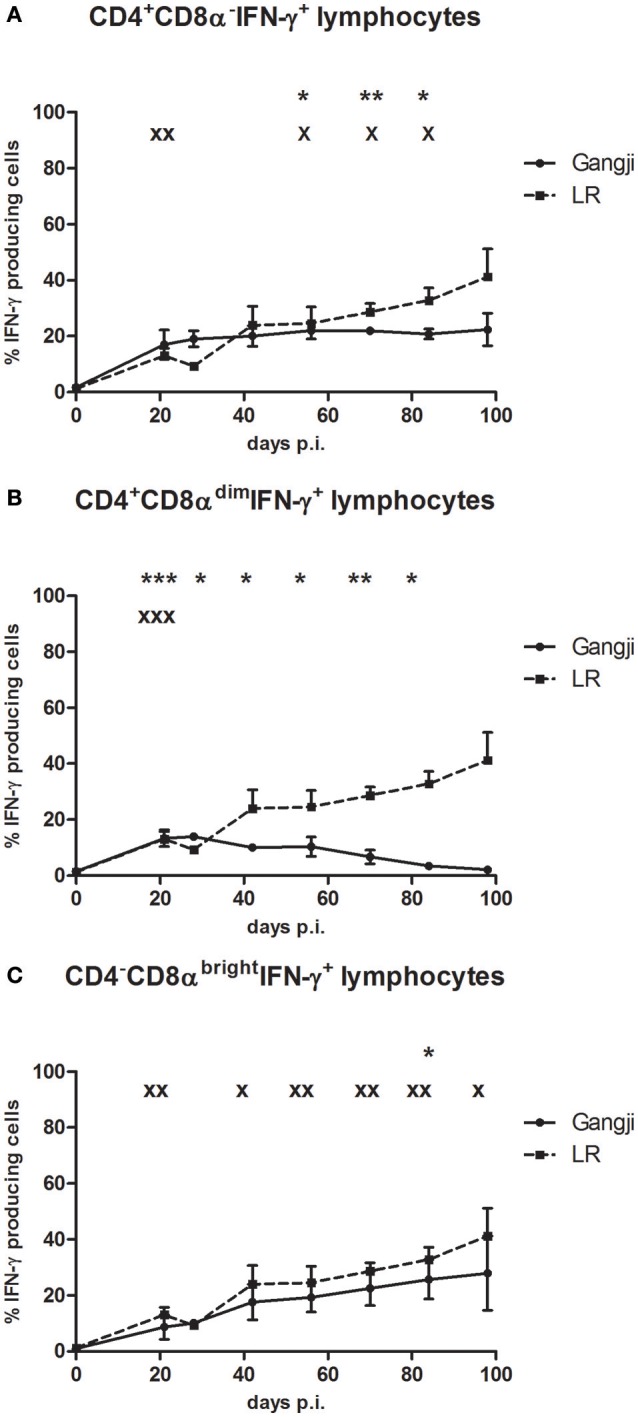
The percentage of IFN-γ^+^ T lymphocyte subsets in PBMCs after a single IPB-G or IPB-LR inoculation. IFN-γ^+^ T lymphocyte subsets in PBMC's of pigs after oral infection with a high dose of the IPB-G (G_high_) or the IPB-LR strain (LR_high_). Cells were restimulated *in vitro* with TLA and demonstrated by flow cytometry following triple staining for IFN-γ, CD3, CD4, and CD8 **(A)**. IFN-γ^+^ cell populations were identified as **(A)** CD3^+^CD4^+^CD8α^−^IFN-γ^+^, **(B)** CD3^+^CD4+CD8α^dim^ IFN-γ^+^, **(C)** CD3^+^CD4^−^CD8^bright^IFN-γ^+^ lymphocytes. The results represent mean percentages ± SD for each group; ^*^ (IPB-G) or x (IPB-LR): *P* < 0.05, ^**^ or xx: *P* < 0.01; ^***^ or xxx: *P* < 0.001 in comparison with day 0.

Results of the above experiment supported our hypothesis that the IPB-G strain reduced the parasite burden. Since many studies suggested an important role for IFN-γ responses in controlling *T. gondii* infections, in a next experiment we compared the kinetics of IFN-γ producing T cell subsets in blood, following infection with the high dose of both strains in an *in vitro* TLA recall assay. Depending on the *T. gondii* strain, differences in the kinetics of circulating IFN-γ producing T cell subpopulations were observed (Figure 8). The CD3^+^CD4^+^CD8α^−^and CD3^+^CD4^+^CD8α^dim^ represent porcine T-helper cells, while CD3^+^CD4^−^CD8α^bright^ cells are cytotoxic T cells (Gerner et al., 2015). Animals inoculated with the IPB-LR strain showed at 21 dpi a significant increase in the CD3^+^IFN-γ^+^ T cell subsets (CD4^+^CD8α^−^, CD4^+^CD8α^dim^, and CD4^−^CD8α^bright^ T cells), with the CD4^+^CD8α^−^ T-helper cells (up to 22.2 ± 8.3% of CD3^+^IFN-γ producing cells) being most prevalent (Figure 8A). This latter population remained stable, whereas the CD4^−^CD8α^bright^ population gradually increased from 9.3 ± 0.87% of the IFN-γ^+^ producing cells at 28 dpi to > 40% (41.1 ± 17.4%) at 98 dpi (Figure 8C). In contrast, the percentage CD4^+^CD8α^dim^IFN-γ^+^ cells gradually decreased from 16.4 ± 1.4% to <2.1 ± 0.8% at the end of the experiment (Figure 8B). In animals inoculated with the IPB-G strain, a similar increase in the percentage of CD4^+^CD8α^dim^IFN-γ^+^ cells at 21 dpi to 10.4 ± 0.4%, and a subsequent gradual decrease to 2.2 ± 0.5% was seen (Figure 8B). The IFN-γ^+^ within CD4^+^CD8α^−^ and CD4^−^CD8α^bright^ T cells gradually increased, reaching significantly higher levels (22.6 ± 11.9% and 18.7 ± 9.5%, respectively) at 84 dpi as compared to 0 dpi (Figures 8A,C). Intriguingly, the increase of the latter T cell population was clearly less pronounced in the G_high_ (*p* < 0.05) than in the LR_high_ group (*p* < 0.01).

**Figure 9:**
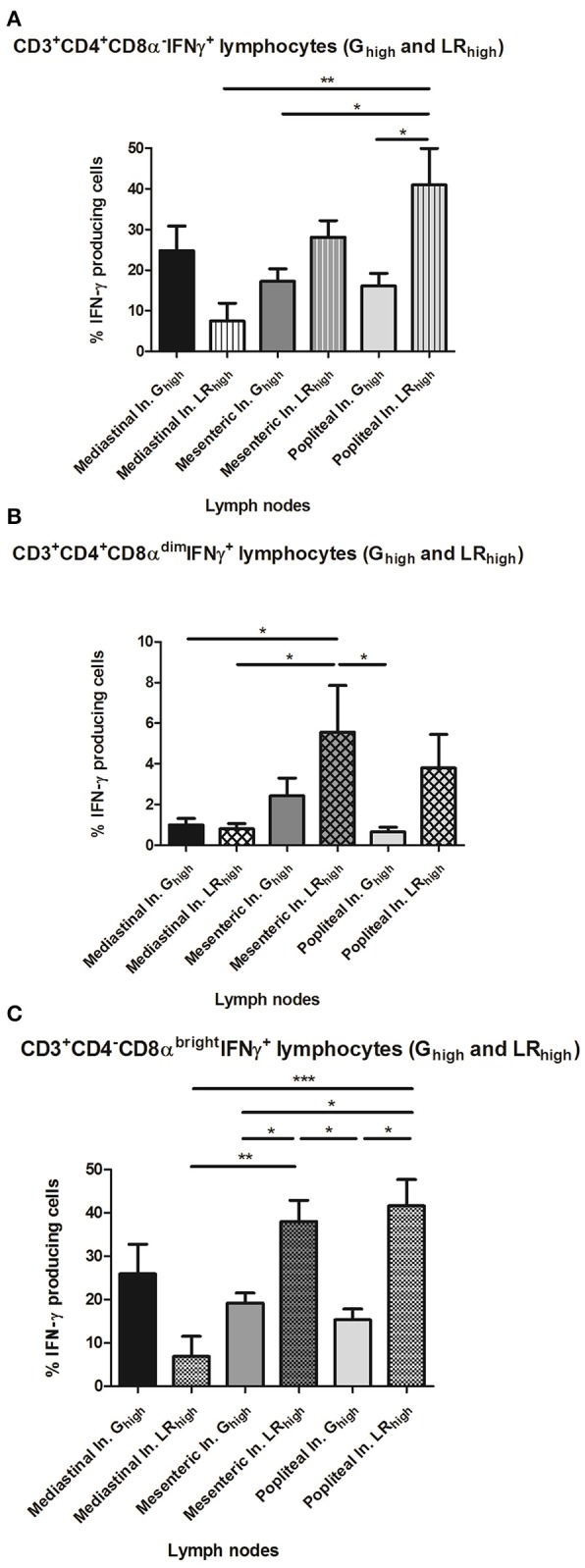
The percentage of IFN-γ^+^ T lymphocyte subsets from lymph nodes after a single IPB-G or IPB-LR inoculation. IFN-γ^+^ T lymphocyte subsets in leukocytes from peripheral lymph nodes of pigs 98 dpi with a high dose of the IPB-G (G_high_) or the IPB-LR strain (LR_high_). Cells were restimulated *in vitro* with TLA and demonstrated by flow cytometry following triple staining for IFN-γ, CD3, CD4, and CD8 **(A)**. IFN-γ^+^ cell populations were identified as **(A)** CD3^+^CD4^+^CD8α^−^IFN-γ^+^, **(B)** CD3^+^CD4+CD8α^dim^ IFN-γ^+^, **(C)** CD3^+^CD4^−^CD8^bright^IFN-γ^+^ lymphocytes. The results represent mean percentages ± SD for each group; ^*^P < 0.05, ^**^P < 0.01; ^***^P < 0.001.

The difference in circulating CD4^−^CD8α^bright^ T cell populations between both high dose groups seems to be reflected on the long term in the significantly higher (*p* < 0.05) percentage of CD4^−^CD8α^bright^ IFN-γ^+^ T cells in the popliteal lymph nodes (LN) in the LR_high_ group than in the G_high_ group at 98 dpi (Figure 9C). Additionally, a similar difference in the percentage of the same population between both groups can be detected in the mesenteric LN. However, in mediastinal LN, which drain heart and diaphragm, a higher percentage of CD4^−^CD8α^bright^ IFN-γ^+^ T cells was found in the G_high_ group, although not significantly different from the LR_high_ group. The distribution of the other T cell subpopulations in different lymphoid tissues shows a comparable pattern: a higher percentage of the CD4^+^CD8α^−^IFN-γ^+^ T was found in the mesenteric (*p* < 0.05) and popliteal (*p* > 0.05) LN of the LR_high_ group than in the G_high_ group (Figure 9A). Likewise for the CD4^−^CD8α^bright^ T cells, a reverse situation was noticed in the mediastinal LN. Regarding the CD4^+^CD8α^dim^IFN-γ^+^ cells, relatively low percentages were detected in both infected groups. The highest counts were found in the mesenteric and popliteal LN in the LR_high_ group, followed by the mesenteric LN in the G_high_ group, whereas they were nearly absent in the other sampled LN (Figure 9B).

## Discussion

In the performed experiments, we compared the single or subsequent infection in pigs inoculated with either a high or a low dose of the IPB-G and the IPB-LR strains for the tissue specific parasite load and the accompanying immune response. The IPB-G strain has a mixed type I/II genotype, while the IPB-LR strain has a classic type II genotype (Jongert et al., 2008; Dubey et al., 2012). The antibody response against GRA7 and TLA, which are frequently used in serological assays in different species, was monitored until 120 dpi to confirm the successful inoculation and persistence of the infection (Figures 2, 3). Overall, for both IgM and IgG, independent from the infection dose, the GRA7-specific antibodies were detected very soon after the initial infection, starting from 10 dpi (Figure 2). Similar to previous results (Verhelst et al., 2015), we detected a late TLA-specific IgG response from 28 to 35 dpi onwards (Figure 3). However, results of the present study demonstrated that strain and dose are important factors to consider, since primary GRA7- and TLA-specific antibody responses could be detected earlier during infection and were higher upon inoculation with a high dose of the IPB-LR strain as compared to the IPB-G strain. A low dose on the other hand resulted in a later and less prominent seroconversion. Interestingly, whereas primary antibody responses were comparable in the heterologous challenge model, a clear IgM response was seen after the challenge with the heterologous strains, indicating exposure to other antigens, presumably due to the genetic diversity of both strains (Figure 5). Burrells et al. (2015) described a significant TLA-specific IgG increase after challenge of pigs with the heterologous strain M4 upon inoculation with the S48 strain. Strikingly, the challenge was performed with oocysts, after an initial inoculation with tachyzoites, stressing the expression of related or identical variability antigens in correlation to the parasite stage and the strain.

Together with a robust humoral response following *T. gondii* infection, a strong innate and cellular immune reaction is well described in mouse and human models, involving several populations of immune cells as well as different activation pathways (Aliberti, 2005; Miller et al., 2009; Andrade et al., 2013; Gazzinelli et al., 2014; Sturge and Yarovinsky, 2014). To date, it is well known that innate immune cells (macrophages, dendritic cells (DC's) and neutrophils) are involved in the acute stage of the infection by triggering the myeloid-differentiation primary response protein 88 (MyD88) signaling pathway after uptake and intracellular recognition of the parasite by CC-receptor 5 (CCR5) or Toll-like receptor (TLR) 11 and 12 in mice; TLR7, 8, and 9 in human and TLR7 and 9 in other mammals like pigs (Miller et al., 2009; Andrade et al., 2013; Koblansky et al., 2013; Gazzinelli et al., 2014). In particular interferon regulatory factor 8 (IRF8)^+^ dendritic cells, activated by the uptake of the parasite's protein profilin, are crucial for the induction of IL-12 secretion in mice. Human and porcine DCs and monocytes are activated by the recognition of the parasite's ssRNA and DNA via TLR7 and TLR9, respectively, prior to their pro-inflammatory cytokine response (Uenishi et al., 2012; Andrade et al., 2013).

Consequently and irrespective of the activated TLRs, the DC-driven IL-12 production leads to the activation of T-helper 1 cells and Natural Killer (NK) cells (Sturge and Yarovinsky, 2014). The latter massively produce IFN-γ, which not only continuously activates macrophages via a positive feedback mechanism, but also elicits the expression of the GTPases. The GTPases family includes four subfamilies: the very large inducible GTPases (VLIG), the Mx proteins, the immunity-related GTPases (IRGs) such as p47 or p65, and the guanylate-binding proteins (GBPs). The p47 IRG offers a robust protection against intracellular pathogens, being recruited to the parasite attachment site at the host cell (MacMicking, 2004; Taylor et al., 2004; Liesenfeld et al., 2011). Subsequently, a lethal damage to the parasitophorus vacuole (PV) is induced, leading to the rupture of the infected cell and release of the parasite into the cytosol (Gazzinelli et al., 2014). The infected cell undergoes necrosis, simultaneously with an enhanced local immune response. It is important to mention that IFN-γ-inducible IRGs are well studied in murine models, where 23 different genes have been identified to date, but the corresponding genes are not present in the human genome, which includes only one gene and one pseudogene (Bekpen et al., 2005; Zhao et al., 2009). This implies that both species deploy other intracellular defense mechanisms against *T. gondii*. The data on the identification of porcine GTPases are scarce, but a high similarity to the human IRGs is mentioned (MacMicking, 2004). Only two porcine GBPs have been reported until now: GBP1 and GBP2, whereas in humans 7 different GBPs have been identified (MacMicking, 2004; Li et al., 2016). More IRG's have been found using Affymetrix GeneChip® Porcine Genome Array but a detailed study in pigs is lacking (Fossum et al., 2014).

Thus, the continuous production of IFN-γ seems to be necessary in maintaining a delicate balance between the host immune system and the parasite's evasion strategies. Additionally, this cytokine plays a pivotal role in controlling both the acute and chronic phase of infection, as it facilitates stage conversion from the tachyzoite to the bradyzoite in acute toxoplasmosis and suppresses the opposite conversion during chronic infection (Denkers, 1999). Likewise, we detected a significant increased IFN-γ production by PBMC's after inoculation with two different *T. gondii* strains (Figure 4), which corroborates our previous results when inoculating pigs with the IPB-G strain (Verhelst et al., 2015). Here, we demonstrated a time- and dose-dependent increase in IFN-γ mRNA expression upon infection with the IPB-G strain. Several studies focused on experimental infection in pigs reported a time-dependent increase of IFN-γ levels in serum, supernatant from cultured PBMCs and IFN-γ mRNA expression in PBMCs and intestinal lymphoid tissues (Solano Aguilar et al., 2001; Dawson et al., 2004, 2005; Verhelst et al., 2015).

On the contrary, the inoculation with the low dose of the IPB-LR strain was almost as potent in inducing a relatively fast and strong IFN-γ production by PBMCs as the high dose of the same strain, which resulted in high IFN-γ mRNA levels at already 2 mpi, that were maintained until 120 dpi. Interestingly, the IFN-γ mRNA production in the LR_high_ dose group did not show any increase over time, implying reaching the maximum capacity from 1 mpi onwards. In line with our findings, IL-12 (IL-12p35 and IL-12p40) mRNA expression was not detected in PBMCs in the acute phase of the infection (7 and 14 dpi) in an earlier study in pigs (Dawson et al., 2005).

Based on the results of the detection of IFN-γ and the parasite DNA in tissues (Table 3), a balance between the host defense mechanisms and the invasion of the parasite was probably established soon after inoculation with the IPB-LR strain. Namely, the high IFN-γ production during the infection study was associated with the high counts of parasite DNA in the animal tissues. On the contrary, in the IPB-G groups IFN-γ production was elevated in the late phase of the infection and resulted in a very low to undetectable parasite load in the tissues, implying that high IFN-γ levels can tip the balance in favor of the host. Intriguingly, based on our observations and unpublished data from the acute infection model with the same strains, we speculate that exposure to a high dose of the IPB-G strain is more effective in activating innate immunity than the low dose of the same strain or the inoculation with the IPB-LR strain. The strain-dependent differences in the IFN-γ production profile may result from the genetic and thus, biological features of the used strains, indicating expression of variable virulence factors toward the intermediate host. In our opinion and according to others (Hunter and Sibley, 2012), the IPB-LR as genotype II strain, activates other pathway than the IPB-G strain, which is of an atypical genotype (mixed genotype I and II). The virulence factors initiating a pathway would be GRA15 (via NF-κB) for the former, and ROP18 (via STAT3/STAT6 pathway) for the latter. Consequently, the IPB-LR induces Th1 type of protective immunity and remains persistent in the chronic phase. When looking at the IPB-LR infected groups, no substantial difference was noticed in the IFN-γ production pattern or in the amount of *T. gondii* DNA at the end of the experiment. In our view, the IPB-LR strain does not show an acute virulence but it is adapted to persist within the intermediate host and, as such, increase own survival. To support these speculations, we observed a much milder clinical manifestation upon inoculation with the IPB-LR strain (Jennes et al., unpublished data). We further hypothesize that resistance to the chronic infection in IPB-G model results from the high acute virulence and the subsequent fast elimination of the tachyzoites before they can successfully multiply and disseminate. Consequently, fewer parasites can survive the initial parasitemia, which eventually will lead to reduced numbers of cysts in the tissues. Importantly, we conducted the studies in a homogenous pig population in order to exclude host diversity; however, as the strains are maintained by serial passage in mice, their virulence might be altered compared to the original isolate. Therefore, it is tempting to speculate that the high IFN-γ production together with the lower parasite counts in the porcine tissues originate from a coevolution toward host tolerance and reduced virulence, as suggested earlier by others (Gazzinelli et al., 2014). Furthermore, looking at the total IFN-γ expression following reinoculation with the heterologous *T. gondii* strain in the second experiment, no obvious difference between the groups could be observed (Figure 6). In the G_high_/LR_high_ group we detected an initial increase, which was followed by a steady decrease after the challenge. The IFN-γ production profile in the reversed infection model (LR_high_/G_high_) supports our previous findings, showing a constant IFN-γ detection over time. Interestingly, in some animals basal or low level of cytokine mRNA were detected at 1mpi, followed by a substantial increase at the later time points, similar to the single infection experiment (Figures 4A, 6A). However, the final IFN-γ concentration at the end of the experiment upon a heterologous challenge was 10 times lower than after a single high dose inoculation. We could speculate that the primary infection with a mixed genotype I/II strain, characterized by a high acute virulence and long-term STAT3 and STAT6 activation, partially modulates the immune response upon the challenge with genotype II strain. As the result, the initial impairment of the Th1 response after the challenge leads to a lower than in a single infection model IFN-γ production, and elimination of a certain fraction of the parasites. Consequently, a reduced amount of the tachyzoites disseminate to convert into bradyzoites. The latter has been shown by a lower parasite load 60 dpi challenge than in IPB-LR experiment (Table 3 and Figure 6).

In regard to the involvement of immune cells in controlling the parasite's dissemination to the tissues and the chronic phase of toxoplasmosis, different populations seem to play a role. As described earlier and analogous to the acute infection stage, the production of IFN-γ is gradually taken over from the innate immune cells by T lymphocytes (Guan et al., 2007). Experimental infections in mice (Jongert et al., 2010; Suzuki et al., 2012) demonstrated the importance of CD4^+^ and CD8^+^ IFN-γ producing T cells in maintaining a chronic *T. gondii* infection, but the exact contribution of each subset remains unknown. Miller et al. (2006) describes higher production of IFN-γ by murine CD4^+^ cells upon *in vitro* stimulation by infected macrophages or by TLA, but admits that the higher protective potential against dissemination of the parasite by CD8^+^ or CD4^+^ lymphocytes is not simply expressed by the amount of this cytokine. Indeed, it seems that IL-4 and IL-10 cytokines, produced by CD4^+^ lymphocytes in addition to IFN-γ, might down regulate this protective capacity against the parasite. In line with that, due to their IFN-γ-independent cytolytic activity, the role of primed CD8^+^ T cells in the host's immunity during chronic toxoplasmosis has been widely acknowledged (Wang et al., 2005; Suzuki et al., 2012; Sa et al., 2013). In pigs, only a few experiments identified CD8^+^ and CD4^+^CD8^+^ cells in the acute phase of the infection as the major source of the IFN-γ production (Solano Aguilar et al., 2001; Dawson et al., 2005). The additive or synergistic effect of CD4^+^ T cells on the activity of the CD8^+^ T cell population should not, however, remain neglected. In our study, regardless of the strain, the CD4^−^CD8α^bright^ T cell subset contained the most IFN-γ positive cells, followed by the CD4^+^CD8α^−^ subset, whereas the CD4^+^CD8α^dim^ T cell subset showed very few IFN-γ positive cells (Figure 8). Additionally, the CD4^−^CD8α^bright^ population showed a temporal increase in IFN-γ production in animals infected with IPB-LR, while the percentage of this subset was rather declining from 4 wpi onwards, when infected with the IPB-G. The IFN-γ production resulting from the induced toxoplasmosis in pigs and the involvement of the different lymphocyte populations are in line with other studies, where the *in vitro* cytokine profile was investigated until 14 (Dawson et al., 2005), 40 (Solano Aguilar et al., 2001), or 56 dpi (Verhelst et al., 2011). However, opposite to the pig model, in murine experiments only two T lymphocyte subsets were differentiated (CD4^+^ and CD8^+^). Furthermore, the extent of the cellular response was positively correlated with the infection dose and the time-interval from the inoculation and was higher when induced by the strain with a greater tissue persistence. In perspective of future experiments in pigs, it would be desirable to focus on other immune cells, involved in the responses throughout the infection such as the immunosuppressive T regulatory (Treg) or Th17 cells. As recently shown in mice, the robust immune reaction expressed by the high IFN-γ levels in the acute phase of the infection severely reduces the activity of Tregs in a IL-2 dependent and IL-10 independent manner (Tenorio et al., 2011; Olguin et al., 2015). In a longitudinal clinical case study of human acquired cerebral toxoplasmosis a dual function of the Treg population was described, by simultaneous down regulation of CD4^+^ and activation of pathogen-specific CD8^+^ T lymphocytes (Rb-Silva et al., 2017). In human congenital infections not only CD4^+^ Treg cell population seems to be involved in the immune reaction triggered by *T. gondii*, but also a different subset of CD4^+^ or CD8^+^, namely Th17. The activity of this population is independent from IFN-γ, IL-4 and perforin activation, as their migration to the inflammation sites in initiated by certain chemokines. Interestingly, the results of the *in vitro* PBMCs stimulation with tachyzoites showed a higher percentage of CD4^+^ IL17 producing cells above the CD8^+^ (Silva et al., 2014). By investigating the fluctuations of the activity of Tregs and Th17 cells during the acute and chronic phase of the infection, we might determine whether these populations are also involved in the persistent immunity toward the parasite.

When considering the parasite load in the tissues and the viability of the cysts in the bio-assay, a clear correlation was found between the amount of detected DNA and the dose of the used strain (Table 3). In general, we observed a decline in the concentration of the parasite's DNA in animals when inoculated with a high dose of the IPB-G, but not the IPB-LR strain. This dose- and time-dependent decline is prominently present in different tissues in the G_high_ group in comparison with G_low_ and G_high1/2t_ groups, indicating that the effect of the high dose is particularly visible after a longer infection time. These results are in line with the findings of Verhelst et al. (2011), where neither parasite DNA nor viable parasites were detected in certain muscle tissues 6 months after initial infection with the IPB-G strain. In the same study, brain and heart of all animals remained infectious as determined by a bio-assay and qPCR. However, these findings are opposite to results obtained in rats and cats, showing that inoculation with increasing amounts of tachyzoites or bradyzoites resulted in a decreased survival rate or in a higher number of tissue cysts, respectively (De Champs et al., 1998; Cornelissen et al., 2014). In addition and similar to our results, others reported a reduction in parasite burden in strain-vaccinated and challenged pigs (Kringel et al., 2004; Garcia et al., 2005; Jongert et al., 2008; Burrells et al., 2015). In these experiments vaccination with oligonucelotides, antigens or infection with attenuated strains can enhance Th1 responses to elicit sufficient protection during the acute phase of the infection, resulting in a lower parasite burden in comparison with the infected control animals. Referring to that, the strains used in our study differ greatly in terms of genetic background and associated virulence, both in mice and in pigs. Therefore, we have grounded scientific reasons to believe that the observed differences in the parasite load upon infection with both strains, especially in the heterologous co-infection model, are not coincidental.

For most tissue samples this dose- and strain-dependent reduction in the number of the tissue cysts in qPCR is consistent with the bio-assay in mice. It is noteworthy that a few qPCR-positive samples were negative in the bio-assay, indicating that parasite DNA, but not viable parasites were present. Consequently, these results show a substantially higher sensitivity of the qPCR method used here above the bio-assay, since it has been optimized and successfully applied for the detection of the parasite in various human or animal tissues, with a detection limit of 2–4 tissue cysts in 100 g of sample (De Craeye et al., 2011). Conversely, both techniques gave positive results on all samples derived from animals upon single inoculation with the IPB-LR strain. Therefore, we can assume that the reduced parasite load occurred due to the earlier infection with the IPB-G strain in the group G_high_/LR_high_. This phenomenon does not seem to be limited to type II strains (Velmurugan et al., 2009; Suzuki et al., 2012), but is also common in type I strains (Burrells et al., 2015).

Summarizing, the groups infected with the IPB-LR strain can serve as a classical model of *T. gondii* persistence in its intermediate host. The prominent production of parasite-specific antibodies, consistent amounts of IFN-γ and activation of cytotoxic T lymphocytes on the one hand and well-distributed DNA concentration together with isolation of the viable parasite on the other hand, clearly prove an established balance between the host immunity and the pathogen's activity. The parasite's persistence appears to be beneficial for the two, under conditions that the immunocompetent host can resist the immunomodulation by *T. gondii*. On the contrary, as the partial or total removal of the tissue cysts was observed in the IPB-G infected animals together with the increasing IFN-γ production profile on both the mRNA and protein level, we propose that the IPB-G strain induces a robust immune reaction in the host in the early phase of the infection. This IFN-γ-mediated response in pigs can lead to the resistance of the host to parasite invasion by elimination of the tissue cysts during the chronic infection. Further experiments to unravel the nature of this resistance are warranted as it could serve an important role in vaccination strategies and in the risk assessment for food safety and human health.

## Ethics statement

This study was carried out in accordance with the recommendations of the ethical standards defined by the EU legislation on the use of laboratory animals (2010/63/EU) and in accordance with the Belgian law (the Royal Decree 29/5/2013 and the Royal Decree 30/11/2001). The protocol was approved by the Ethical Committee of the faculties Veterinary Medicine and Bioscience Engineering at Ghent University (approval number no. 176 2009/149) and by the Ethical Committee of the WIV-ISP Institute (approval no. 176 20140704-01). The Biosafety Level 2 permit for working with pathogens for Ugent, Merelbeke: AMV/11062013/SBB219.2013/0145. The Biosafety Level 2 permit for working with pathogens for WIV-IPH, Brussels: 415240.

## Author contributions

MJ designed the study, acquired and analyzed the data and drafted the manuscript. SD designed the study, acquired, and analyzed data and revised the manuscript. BD helped to design the flow cytometry study and revised the manuscript. PD and KD helped to design the study, gave valuable input and revised the manuscript. EC designed the study, analyzed the data and wrote the manuscript.

### Conflict of interest statement

The authors declare that the research was conducted in the absence of any commercial or financial relationships that could be construed as a potential conflict of interest.
