# Supplementary material for: Strain- and Dose-Dependent Reduction of Toxoplasma gondii Burden in Pigs Is Associated with Interferon-Gamma Production by CD8+ Lymphocytes in a Heterologous Challenge Model
Source: Front Cell Infect Microbiol. 2017 Jun 8;7:232. doi: 10.3389/fcimb.2017.00232 (PMC5462990; doi:10.3389/fcimb.2017.00232)
Supplement: Supplementary file 1 [file Image1.PDF]

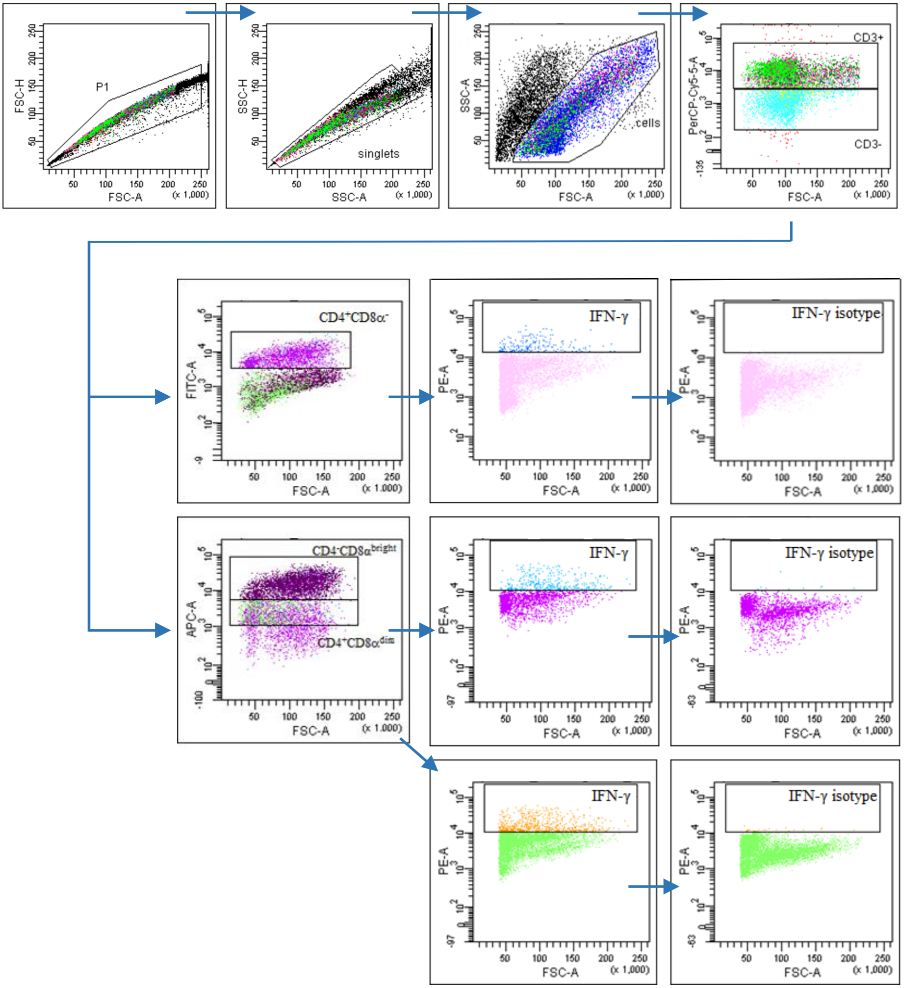

Appendix 1

The gating strategy for the flow cytometric detection of IFN-γ^+ T lymphocytes. The IFN-γ^+ cell populations were identified as single cell $CD3^+$ cells (upper panel) and then divided in $CD4^+CD8\alpha^-$IFN-γ^+, $CD3^+CD4^-CD8^{bright}$ IFN-γ^+ and $CD3^+$CD4+$CD8\alpha^{dim}$ IFN-γ^+ lymphocytes (lower panel).
